# Glacial Refugia in Pathogens: European Genetic Structure of Anther Smut Pathogens on *Silene latifolia* and *Silene dioica*

**DOI:** 10.1371/journal.ppat.1001229

**Published:** 2010-12-16

**Authors:** Elodie Vercken, Michael C. Fontaine, Pierre Gladieux, Michael E. Hood, Odile Jonot, Tatiana Giraud

**Affiliations:** 1 Université Paris-Sud, Laboratoire Ecologie, Systématique et Evolution, UMR 8079, Orsay, France; CNRS, UMR 8079, Orsay, France; 2 Department of Biology, Amherst College, Amherst, Massachusetts, United States of America; University of Melbourne, Australia

## Abstract

Climate warming is predicted to increase the frequency of invasions by pathogens and to cause the large-scale redistribution of native host species, with dramatic consequences on the health of domesticated and wild populations of plants and animals. The study of historic range shifts in response to climate change, such as during interglacial cycles, can help in the prediction of the routes and dynamics of infectious diseases during the impending ecosystem changes. Here we studied the population structure in Europe of two *Microbotryum* species causing anther smut disease on the plants *Silene latifolia* and *Silene dioica*. Clustering analyses revealed the existence of genetically distinct groups for the pathogen on *S. latifolia*, providing a clear-cut example of European phylogeography reflecting recolonization from southern refugia after glaciation. The pathogen genetic structure was congruent with the genetic structure of its host species *S. latifolia*, suggesting dependence of the migration pathway of the anther smut fungus on its host. The fungus, however, appeared to have persisted in more numerous and smaller refugia than its host and to have experienced fewer events of large-scale dispersal. The anther smut pathogen on *S. dioica* also showed a strong phylogeographic structure that might be related to more northern glacial refugia. Differences in host ecology probably played a role in these differences in the pathogen population structure. Very high selfing rates were inferred in both fungal species, explaining the low levels of admixture between the genetic clusters. The systems studied here indicate that migration patterns caused by climate change can be expected to include pathogen invasions that follow the redistribution of their host species at continental scales, but also that the recolonization by pathogens is not simply a mirror of their hosts, even for obligate biotrophs, and that the ecology of hosts and pathogen mating systems likely affects recolonization patterns.

## Author Summary

Global change is expected to cause the large-scale redistribution of species, including pathogens that threaten the health of domestic populations and natural ecosystems. Predicting the dynamics of invasive pathogens is therefore a major challenge for the 21^st^ century ecologists and epidemiologists. Because past climatic events have shaped the current distribution of species, the study of migration of pathogens during glaciation cycles provides insights into the constraints upon pathogen spread. We studied the structure of European populations of two pathogenic fungal species infecting two species of wild plants. The pathogen with the host's broadest ecological tolerance has followed the postglacial colonisation pathway of its host from the major refugia in southern Europe, although the pathogen likely persisted in more fragmented refugia. The other pathogen showed less clear-cut genetic patterns and some evidence of possible northern refugia, in agreement with the host's more fragmented distribution and ecological preference. These results indicate that pathogen invasions are likely to follow large-scale migration events of their host species in response to climate change, but also that the recolonization by pathogens is not simply a mirror of their hosts, and that the correlation between the colonization patterns of hosts and pathogens depends on host ecology.

## Introduction

Understanding the dynamics of emerging infectious diseases and their routes to colonize new geographic regions is a major challenge for ecologists in an effort to prevent negative impacts upon human, domestic, and natural populations. Because pathogens causing emerging diseases have not coevolved with the host or the ecosystem in which they emerged, they may be more likely to pose a threat to biodiversity through biomass loss and extinction of host species than those responsible for endemic diseases [1]. In recent years, concerns about emerging diseases have been increasing in light of the first evidence of a current period of global climate change. Indeed, the increase of average temperatures in many areas of the world is thought to promote the expansion of exotic pathogens [2]. In particular, invasion by fungal pathogens is a major concern in agricultural and biodiversity management, as they infect many crops and wild plants [3], [4]. About 30% of emerging infectious diseases of plants are caused by fungi, and the change of environmental conditions is thought to be the major driver of fungal invasions [1] and disease outbreaks [5]. Fungal disease outbreaks in humans have also been suggested to be linked to climate change [6], [7]. Warmer and wetter conditions favour the growth and transmission of fungal pathogens, and host shifts often occur in conjunction with episodes of global climate change [8]. Based on current trends, emerging infectious diseases caused by fungal pathogens are likely to increase in the near future, with significant severe ecological, economic and social consequences.

One way to predict the invasion routes and dynamics of emerging infectious diseases in response to current climate warming is to study the past migrations of pathogens and their hosts during historic periods of climate changes. During the last glacial maximum, the Arctic ice sheet extended into a large part of Europe and limited the survival of most organisms into Southern Mediterranean refugia (i.e., Iberia, Italy and the Balkans [9], [10]). As the climate warmed and the ice sheet retreated, many species that had persisted inside the glacial refugia experienced massive migrations into the newly available temperate territories [11]. Such processes are expected to lead to a strong, large-scale geographic structure of genetic variation, consistent with what is observed in many widespread European plants (e.g. [12]–[17]). Genetic differences between spreading populations are likely to result from both natural selection and stochastic processes in small populations. Successive founder events during the process of range expansion are expected to lead to a loss of variation and further divergence between lineages derived from different refugia [9], [18]–[21].

Few studies have investigated whether the population structures of pathogens have also been impacted by the glaciations in Europe (but see e.g. [22]–[25]). In the case of host-pathogen systems, comparative phylogeography can also provide insights into host and pathogen co-evolutionary histories and identify causal factors determining their combined distributions [26]–[28]. In fact, pathogen populations are often more differentiated than their hosts, and the study of pathogens can complement or improve our knowledge on the host population genetic structure [29]–[37]. The extent to which the phylogeographic structure of pathogen populations mirrors that of the host depends on the degree of specificity and the obligate nature of pathogenic interaction [38]. A significant co-structure between the populations of the host and the pathogen suggests that the distribution and migration of the host impose a major constraint on the distribution of the pathogen [8]. On the other hand, the absence of congruence in population structure is consistent with independent host and pathogen colonization routes. In addition to pathogen specialization, the hosts' niche breadth and demographic characteristics may affect the persistence of disease and opportunities for host range expansion during large-scale migrations that follow climate change [39]–[41]. Thus, an approach that integrates knowledge of host and pathogen biology is essential to many theoretical and applied issues related to disease emergence in response to climate change.

*Microbotryum violaceum sensu lato* is a species complex of basidiomycete fungi responsible for anther smut disease in many plants in the Caryophyllaceae. These fungi are obligate pathogens that sterilize their hosts. Infected plants contain fungal teliospores in place of the pollen and female structures do not mature; female plants in dioecious species also develop spore-bearing anthers. Teliospores are transmitted from diseased to healthy plants mostly by insects that normally serve as pollinators. Therefore, the dispersal routes of the host's pollen and of the pathogen's spores are constrained by the same vectors. Plants also disperse by seed, while the fungus is not vertically transmitted, resulting in higher genetic differentiation in the pathogen than in the host [42]. The sibling species encompassed in *Microbotryum violaceum sensu lato* [43]–[49] show strong host specificity [45], [47], [50]. The most widely studied species are *Microbotryum lychnis-dioicae* [51] (called MvSl in [46] and hereafter) and *M. silenes-dioicae* [51] (called MvSd in [46] and hereafter), which infect respectively *Silene latifolia* (white campion) and *S. dioica* (red campion). These two closely related host-pathogen systems are interesting models for studying the combined demographic histories of pathogens and their hosts because (i) populations of this pathogen are more differentiated than those of its hosts [42], (ii) the fungus is completely dependent on its hosts and the same vectors disperse the fungus and the host pollen, (iii) these *Microbotryum* species are highly host specific in the field ([52]), (iv) *Silene* and *Microbotryum* species have similar generation times (one per year [53], [54]), (v) there has been no attempt to control the disease because it affects plants without economic interest, and (vi) *Silene* and *Microbotryum* are model organisms for a variety of topics in ecology and evolution, with therefore numerous studies available on their life-history and ecology [55].

Recent studies on the phylogeographic history of the two host plants showed genetic evidence of post-glacial recolonization from Mediterranean refugia. In *S. latifolia*, analyses of chloroplast DNA (cpDNA) polymorphism showed clearly structured haplotype variation in Europe, with haplotypes from Eastern and Western Europe forming divergent groups descended from haplotypes currently distributed in southern Europe, and in particular from the Iberian and Balkan Peninsulas [56]. The phylogeography of *S. dioica* in Europe has been less well studied, although the pattern of cpDNA polymorphism was also suggestive of post-glacial recolonisation from multiple refugia [57], [58]. A goal of the current study was therefore to determine the extent to which population structure of *Microbotryum* species parasitizing *S. latifolia* and *S. dioica* showed similar patterns of post-glacial history. We also investigated whether life history differences between the two host species constrained the distributions of the pathogens within the host migration pathways. *S. latifolia* and *S. dioica* differ with respect to their ecologies, which might strongly impact the genetic structure and diversity of their specialized pathogens. *S. latifolia* has an extensive range and occurs in most of Europe, as well as in Middle Asia and the Steppe area of south Siberia [59]. This plant is found mainly in open areas, such as hedgerows and in arable fields, therefore often experiencing extinction-recolonisation events in frequently disturbed habitats. In contrast, the distribution of *S. dioica* covers mainly Central, Northern, and Western Europe [59], but not the Mediterranean regions. This plant is found in meadows, cliffs, moist forest, and mown pastures at higher elevations, preferring colder and more humid habitats than *S. latifolia*, and experiencing more stable population dynamics [59]–[62]. It has been suggested that these differences in host life history have affected the distribution of genetic diversity at a small geographical scale in the pathogen species, with lower microsatellite variation and higher differentiation among populations in the anther smut fungus parasitizing *S. latifolia* than in the fungus parasitizing *S. dioica* [63]. It is likely that the phylogeographic structure of the European populations of the two pathogen species will also be influenced by differences in the dynamics of the host-pathogen systems.

We therefore determined the population structures of the *Microbotryum* species infecting *S. latifolia* and *S. dioica* using microsatellite markers in order to address the following specific questions: (i) Do the phylogeographical structures of the *Microbotryum* species show signatures of post-glacial recolonisation of Europe, and in particular from Southern refugia? (ii) do the population structures of the two pathogens differ from each other, and if so, are these differences consistent with expectations based upon known ecological differences of their plant hosts? (iii) are the phylogeographic patterns of *Microbotryum* species comparable at a continental scale to those of their respective hosts?

## Results

### Genetic polymorphism in *Microbotryum lychnidis-dioicae* (MvSl) and *M. silenes-dioicae* (MvSd)

Among *Microbotryum* samples collected on *S. latifolia* and on *S. doica* across Europe (Figure S1), analysis of variation at 11 microsatellite markers revealed that both pathogen species displayed much lower levels of heterozygosity than expected under Hardy-Weinberg Equilibrium (HWE). The dataset included 701 MvSl individuals from 187 localities and 342 MvSd individuals from 68 localities, where hybrids and cross-species disease transmission between MvSl and MvSd identified in a previous study [52] were removed from the dataset. Descriptive statistics on the polymorphism of MvSd and MvSl and on deviations from HWE are shown in Table 1 and additional details are given in Text S1 and Tables S1 and S2. MvSl for instance exhibited only 3% of heterozygous genotypes while 73% were expected under HWE, which is consistent with the high selfing rates previously reported in *Microbotryum*. One marker, SL19, showed extreme *F_IS_* values in MvSl, between −1 and 1, and was almost fixed in the heterozygous state in MvSd (Table 1). Analyses were therefore performed with and without this marker for subsequent analyses, but the results were highly similar.

**Table 1:** Summary statistics on the 11 microsatellite loci in *Microbotryum lychnidis-dioicae* (MvSl) and *Microbotryum silenes-dioicae* (MvSd).

Locus	Repeat	*Microbotryum lychnidis-dioicae* (MvSl)					*Microbotryum silenes-dioicae* (MvSd)				
		N	Range	A	H_o_/H_e_	F_IS_	N	Range	A	H_o_/H_e_	F_IS_
E14	(AG)_15_	670	7–23	11	0.03/0.74	0.86***	329	9–15	4	0.02/0.31	0.93***
E17	(AG)_20_	649	2–17	15	0.01/0.86	0.90***	326	1–5	5	0.01/0.76	0.98***
E18	(AG)_15_	535	4–25	20	0.01/0.88	0.98***	273	9–24	14	0.00/0.89	0.99***
SL5	(CT)_18_	678	11–22	12	0.00/0.82	0.98***	329	12–18	7	0.00/0.82	0.99***
SL8	(GA)_15_	534	1–10	10	0.01/0.63	0.96***	303	1–5	3	0.02/0.15	0.84***
SL9	(CT)_9_	683	3–14	12	0.04/0.83	0.89***	339	6–10	5	0.02/0.68	0.98***
SL12	(GT)_10_	680	1–7	7	0.03/0.64	0.73***	314	3–6	4	0.06/0.53	0.89***
SL19	(AAC)_3_AAA(AAC)_12_	662	1–9	9	0.10/0.84	0.29***	321	1–7	7	0.86/0.63	−0.36^+++^
SVG5	(TG)_8_	695	2–9	7	0.09/0.68	0.59***	318	4–9	5	0.09/0.56	0.83***
SVG8	(GT)_12_	679	9–25	15	0.02/0.87	0.89***	337	9–23	10	0.04/0.82	0.95***
SVG14	(CTC)_2_(TTC)_10_	682	6–18	7	0.02/0.24	0.63***	318	14–22	6	0.05/0.70	0.92***
all		649.7 (±58.2)		11.4 (±4.1)	0.03/0.73		318.8 (±18.4)		6.4 (±3.2)	0.11/0.62	

Repeat: Motifs and repeat numbers of the microsatellites in the isolated clones that served to develop the markers. N: number of samples successfully genotyped; range: allelic size range in number of repeats; A: number of alleles observed; H_o_: observed heterozygosity and He the expected heterozygosity (or overall genetic diversity in Nei 1987). Symbols (***) and (^+++^) show significant (p<0.001) deficit or excess in heterozygosity compared to Hardy-Weinberg expectations. A more detailed interpretation of the genetic polymorphism in MvSl and MvSd is provided in Text S1.

### Genetic structure and genetic diversity in *M. lychnidis-dioicae* (MvSl)

For MvSl, multiple estimates of the genetic structure at the European scale showed the existence of at least three to five strongly supported clusters, i.e. populations genetically differentiated from each other. We used the model-based Bayesian clustering algorithms implemented in STRUCTURE, InStruct and TESS. The program STRUCTURE assumes a model with K clusters, each of which being characterized by a set of allelic frequencies. Assuming HWE and linkage equilibrium among loci within clusters, the program estimates allelic frequencies in each cluster and the proportion of ancestry from the different clusters in each individual. The program InStruct is an extension of the approach implemented in STRUCTURE, relaxing the assumption of HWE within clusters. InStruct instead jointly estimates selfing rates and individual membership on the basis of selfing rates, and is therefore well suited to selfing organisms such as *Microbotryum*. TESS is another extension of STRUCTURE, incorporating a spatial component into the clustering algorithm, so that geographically closer individuals are a priori more likely to belong to the same cluster. This may help revealing subtle geographical structure [64]. We attempted to identify the number of clusters (K) that best described the population structure using (1) the probability of the data under the considered value of K, i.e. Ln(Pr(X|K)), and its rate of change when increasing K; and (2) the Deviation Index Criterion (DIC), i.e. a model-complexity penalized measure of how well the model fits the data.

For MvSl, the programs STRUCTURE and InStruct showed that values of DIC decreased and LnP(X|K) increased from K = 1 to 10 (Figures S2a–c), indicating that increasing the number of clusters continuously improved the fit of the model to the data. However, the variation in LnP(X|K) showed a marked break at K = 5 in STRUCTURE analyses, with a much weaker increase of probability with increasing K afterwards (Figures S2a and b). The inclusion of space in the clustering modelling, as implemented in TESS 2.3, resulted in minimal DIC values at K = 5 and K = 6 (Figure S2d). Increasing K above 5 may therefore add little information for understanding large-scale population structure in Europe, although it would likely reveal a genuine population structure, relevant at smaller scale.

The admixture proportion (*α*) between clusters was low, as shown by the mean *α  = *0.033±0.000 over all the runs between K = 2 to 15 (10 replicates for each K) in the STRUCTURE analysis. This indicates that most of the genotypes are drawn from a single cluster, with little admixture among clusters (see Figure S3 for K = 5). There is therefore almost complete lack of gene flow among clusters.

Replicates conducted for each of the three algorithms showed dominant and minor modal solutions for membership probabilities (Figure S4). However, the dominant clustering solutions recovered from the three analyses (InStruct, STRUCTURE and TESS) were highly similar (see Figure S3 for K = 5). The three methods were therefore congruent in their inference of the population structure of MvSl in Europe. The differences between the dominant and minor modal solutions most often corresponded to a genetic structure appearing at higher K values (Figure S4). For instance, the Italian cluster was assigned to the Eastern group at K = 2 in the dominant solution, and to the Western group in other simulations.

**Figure 1:**
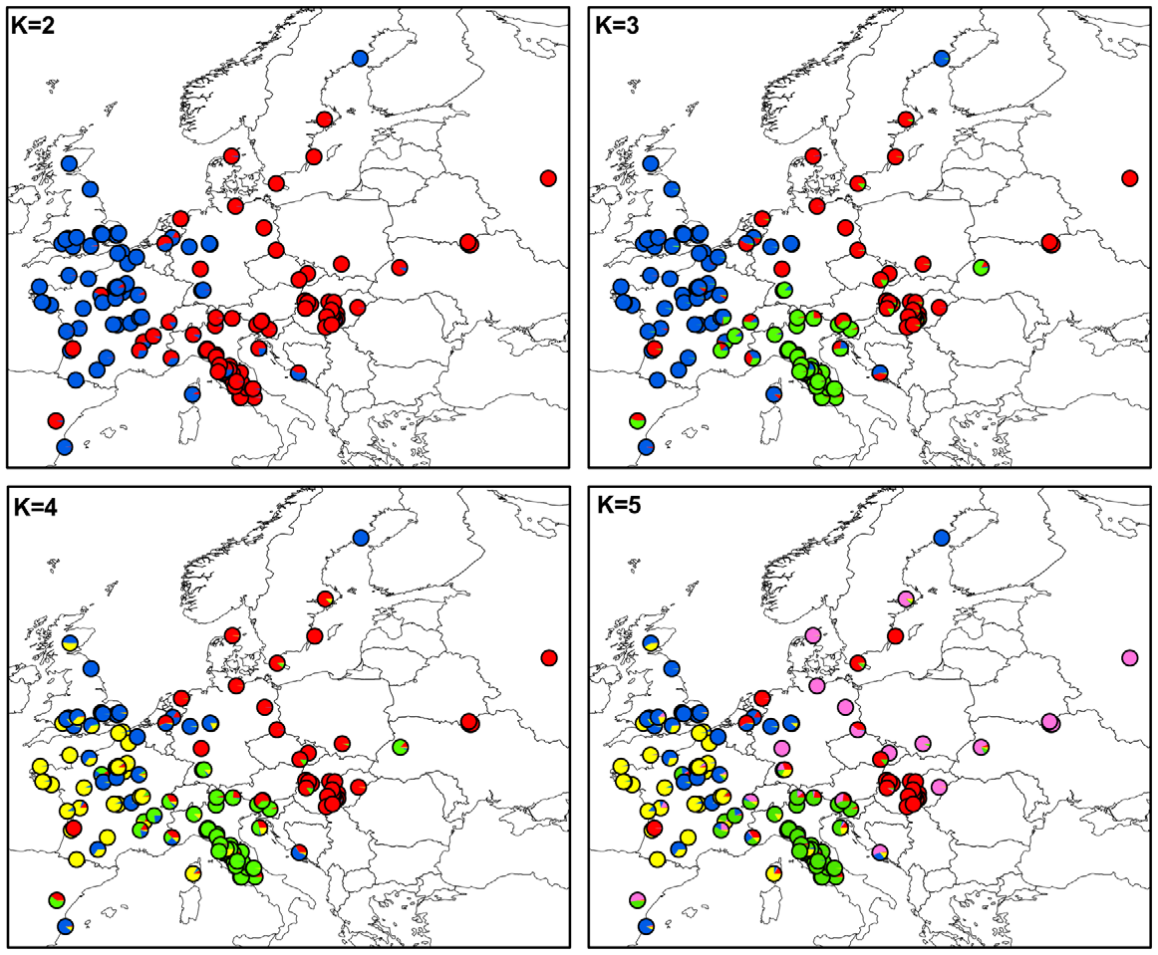
Maps of mean membership probabilities per localities from the InStruct analysis for *Microbotryum lychnidis-dioicae* (MvSl) assuming 2 to 5 clusters.

Figure 1 shows the maps of mean membership probabilities per locality for MvSl genotypes from the InStruct analysis for K = 2 to 5. At K = 2, the analyses revealed a clear West-East partitioning. Simulation of a third cluster separated the Italian genotypes from the Eastern group. At K = 4, the Western cluster was subdivided into two clusters, one with a more northern distribution (blue, called hereafter Northwestern and abbreviated as *Nwestern*) and the other more to the south (yellow, called hereafter Southwestern and abbreviated as *Swestern*). At K = 5, the Eastern group splitted into two clusters, one bordering the Balkan peninsula (red, called hereafter the *Balkan* cluster) and one spreading toward Eastern Europe and Russia (purple, called hereafter the *Eastern* cluster). When increasing K, further clusters were identified, without evidence of admixture, and corresponding to more local geographical regions: for instance the UK became isolated, and then the most eastern part of Europe (Figure S4).

We also applied a Principal Component Analysis (PCA) on the microsatellite allele frequencies, which is a multivariate approach that does not rely on any model assumptions. It instead transforms a number of possibly correlated variables into a smaller number of uncorrelated components, the first principal components accounting for as much variability in the data as possible. The PCA fully recovered the population structure inferred by the three Bayesian clustering methods, as shown by the first four PCs, which explained 35% of the total variance in allelic frequencies (Figures S5 and S6).

The clusters displayed large and significant differences in allelic frequencies (global F_ST_ = 0.38, 95% CI: [0.29–0.46], P<0.001 for all pairs of clusters). The *Nwestern* and *Swestern* clusters showed the lowest differentiation (F_ST_ = 0.24) while the *Balkan* and *Swestern* clusters were the most different (F_ST_ = 0.47). The clusters also differed significantly with respect to their genetic diversities (Table 2), with the *Italian* cluster displaying significantly higher gene diversity (He) than the *Balkan*, *Eastern* and *Nwestern* clusters (Wilcoxon Signed Rank (WSR) tests, P = 0.008, P = 0.033, and P = 0.026, respectively, Table 2). The *Balkan* cluster exhibited a significantly lower allelic richness (number of alleles controlling for differences in sample size) than the *Eastern* and *Italian* clusters (P = 0.016 and P = 0.003, respectively), while the others had intermediate values.

**Table 2:** Genetic polymorphism and spatial pattern within each cluster of *Microbotryum lychnidis-dioicae* (MvSl) and *Microbotryum silenes-dioicae* (MvSd).

	*Microbotryum lychnidis-dioicae* (MvSl)					*Microbotryum silenes-dioicae* (MvSd)	
Cluster	NWest_B (K3)	SWest_J (K5)	Italian (K4)	Balkan (K1)	Eastern (K2)	K1	K2
**Microsatellite polymorphism**							
N	130	147	132	93	84	219	122
Ar ± SE	3.6±0.6	3.8±0.7	5.0±0.5	3.3±0.5	4.4±0.6	4.5±0.9	4.6±0.6
pAr ± SE	0.58±0.25	0.60±0.20	1.17±0.29	0.47±0.18	0.89±0.35	1.43±0.42	1.57±0.42
He	0.46±0.26	0.45±0.32	0.63±0.13	0.35±0.27	0.43±0.28	0.48±0.30	0.55±0.18
F_IS_	0.85	0.90	0.95	0.93	0.96	0.74/0.91	0.86/0.96
Selfing rate	0.93	0.95	0.94	0.91	0.92	0.92	0.94
**Spatial pattern**							
Ln(dist_F(1)_)	3.53	4.32	3.06	2.17	6.52	6.43	2.24
n_F(1)_	245	285	655	16	176	1931	1082
Sp	0.17	0.02	0.06	0.29	0.01	0.06	0.15
b	−0.070	−0.013	−0.037	−0.113	−0.004	−0.037	−0.089
P-value	<0.001	0.115	0.004	<0.001	0.407	<0.001	<0.001

N: within-cluster samples size; Ar: Allelic richness; pAr: private allelic richness; He: expected heterozygosity; Selfing rate: as inferred by InStruct; the first value of F_IS_ for MvSd is when including the marker SL19 and the second value without this marker; Ln(distF(1)) and nF(1): logarithm of the mean geographic distance between genotypes of the first distance class and number of pairs considered in this class; spatial Sp statistic within cluster; b, regression slope between the kinship coefficient and the logarithm of the geographic distance; Mantel test's P-value (H_0_: b_obs_ = b_exp_; H_1_ = b_obs_<b_exp_).

Within clusters, significant isolation by distance (IBD) was detected in the *Balkan*, *Nwestern*, and *Italian* clusters (Table 2), indicating that genetic differentiation increased with geographic distance in these clusters. No significant IBD was detected within the *Swestern* and *Eastern* clusters (Table 2). The level of spatial structure was quantified by the *Sp* statistic, which accounts for variation in sampling intensities; high values of *Sp* are indicative of low population density and/or limited dispersal [65]. *Sp* values were close to 0 within the *Italian*, *Swestern*, and *Eastern* clusters, but were much higher within the *Balkan* and *Nwestern* clusters (Table 2).

Within-cluster selfing rates estimated from InStruct analyses were extremely high (s = 0.91±0.03 on average), in agreement with previous studies and with the high F_IS_ values within clusters (Table 2).

**Figure 2:**
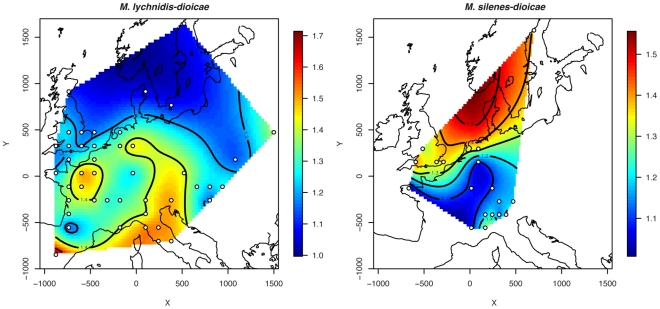
Map of allelic richness overall loci. Samples have been aggregated on a grid in order to make this calculation on a minimum sample size of at least 4 genotypes. Localities where the sample size was below this threshold were not considered.

A European spatial map of genetic diversity was generated by aggregating geographically close samples together on a grid, considering only grid cells where the sample size was higher than 4. The interpolated values of allelic richness showed that genetic diversity increased in the southward direction, with the highest value observed in the Italian and Iberian peninsulas and the lowest values in northern Europe (Figure 2). Such a latitudinal trend was confirmed by the highly significant negative correlation observed between latitude and allelic richness (r = −0.57, P<0.0001); no significant correlation was found between longitude and allelic richness (r = 0.036, P = 0.892). High genetic diversities were also observed in the northern half of France and along a longitudinal line separating the Eastern and Western parts of Europe (Figure 2).

**Figure 3:**
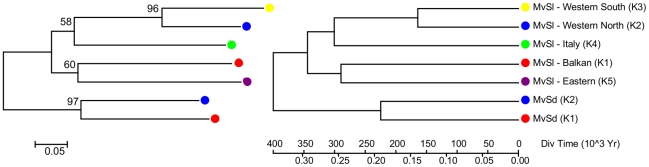
Microsatellite distance-based Neighbour-Joining trees on intraspecific clusters for both *Microbotryum lychnidis-dioicae* (MvSl) and *M. silenes-dioicae* (MvSd). The root of the trees was placed on the branch separating the two species. The bootstrap values above 50% are shown.

We analyzed the relationships among clusters using neighbour-joining population trees, respectively based on Nei's D_A_ distance, shared allele distance D_SA_, Chord's distance and Goldstein's (δµ)^2^ distance. As the different trees provided similar topologies, only the tree based on Nei's D_A_ distance is presented (Figure 3). The trees suggested that the *Eastern* groups would have diverged first, followed by the Italian cluster and then by the Western groups. The Eastern and Western group would then have further split into two clusters each. Rough estimate of separation time between clusters can be deduced from distances between clusters assuming that the divergence between the two species occurred 400,000 yr BP [52] and assuming clocklike evolution of microsatellite markers [66]. The separation of the 5 clusters can thus be roughly estimated to have occurred between 200,000 and 350,000 yr BP (Figure 3).

### Genetic structure, genetic diversity and selfing rates in *M. silenes-dioicae* (MvSd)

**Figure 4:**
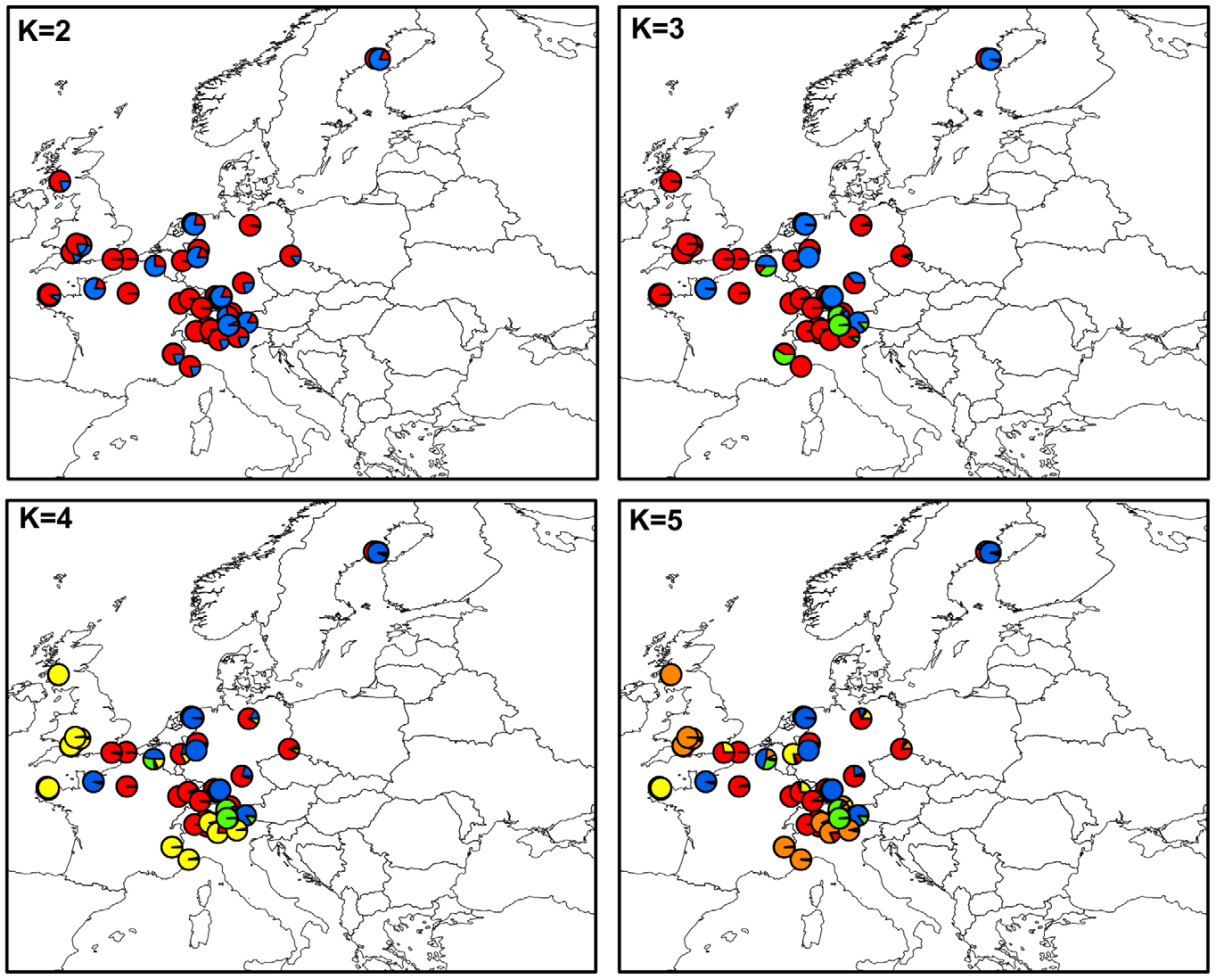
Maps of mean membership probabilities per locality from the InStruct analysis for *Microbotryum silenes-dioicae* (MvSd), assuming 2 to 5 clusters.

In MvSd, multiple estimates of the genetic structure at the European scale provided confidence in existence of several distinct clusters. As for MvSl, the three Bayesian clustering analyses (InStruct, STRUCTURE and TESS) all indicated that DIC decreased and LnP(X|K) increased with increasing K (Figure S7). Again, genotype assignment probabilities were always very high, with very little admixture among clusters (mean α = 0.030±0.002 over the 140 runs from K = 2 to 15 simulated clusters; Figure S8), and were similar for the three algorithms used (data not shown). The spatial distributions of the two clusters identified at K = 2 appeared highly intermingled, with however a slight West-East trend of separation. Further clusters differentiated as K increased, but without any obvious large-scale geographical pattern (Figure 4). Similar genetic partitioning was recovered using PCA (Figure S9). The first PC accounted for 20% of the variance in the allelic frequencies and clearly separated genotypes into the same two groups as those identified using Bayesian clustering approaches at K = 2 (Figure S9). The differences in allelic frequencies between them were high, with a F_ST_ value of 0.34 (95% CI: 0.17–0.49). The successive PCs accounted for less than 11% of the total variance in allelic frequencies each and revealed the same clusters of genotypes as those observed in Bayesian clustering analyses (Figure S9).

The two clusters identified at K = 2 represented the only structure with a large-scale geographical pattern. The F_ST_ values between sites where the sampling was higher or equal to 10 samples showed that a very high level of genetic differentiation between clusters was observed, even in the regions where populations from different clusters were intermingled (Figure S10). Within clusters, there was no significant IBD, i.e. no significant increase in F_ST_ with geographic distance (Mantel test, P = 0.418). The two clusters showed a spatial genetic structure of a similar level to that observed in MvSl, with *Sp* values of 0.06 and 0.15 for the clusters 1 and 2, respectively (Table 2).

Selfing rates within each of the two clusters, inferred from Instruct, were high and similar to those in MvSl (mean selfing rates at K = 2: 0.93±0.01), consistent with the high F_IS_ values (Table 2). The two clusters of MvSd showed genetic diversities comparable to those observed in MvSl (Table 2), and did not differ significantly from each other with respect to gene diversity (WSR test, P = 0.424) and allelic richness (WSR test, P = 0.594). In contrast to MvSl, spatially interpolated values of allelic richness increased northwards (Figure 2), although the correlation with latitude was significant only at a marginal level (r = 0.43, P = 0.073). The correlation with longitude was not significant (r = −0.008, P = 0.975).

## Discussion

### Mating system and local population structure

Very high selfing rates were inferred in both *Microbotryum* species (s = 0.91 for MvSl and s = 0.93 for MvSd), in agreement with the high deficits in heterozygotes (Text S1, Table 1). *Microbotryum* species are in fact known to have a selfing mating system [67], [68], but the estimations of selfing rates in natural populations inferred here are more precise and even higher than previously thought [67]. These high selfing rates appear to result both from an intrinsic preference for intra-tetrad mating [69] and from lack of outcrossing opportunities when the spores are deposited on a new host plant. The lack of outcrossing opportunity is supported by the observation that selfing rates under choice experiments (when given the opportunity to self or outcross on plants) are lower (ca. 0.70 [70]) than those inferred here in natural populations.

The marker SL19 showed extreme *F_IS_* values in MvSl and was almost fixed in the heterozygous state in MvSd. This was not particularly surprising given that *Microbotryum* species undergo mostly intra-tetrad mating, which can lead to an excess in heterozygosity in regions of the genome near the centromeres and on the sex chromosomes carrying the mating type locus [69], [71]. Because the mating type segregates at the first meiosis division, intra-tetrad mating automatically restores heterozygosity in all regions linked to centromeres and linked to the mating type locus, as they also segregate at the first meiotic division [69], [71].

In addition to selfing, the study of local genetic structure and diversity for both *Microbotryum* species across Europe revealed patterns consistent with the dynamics of a metapopulation [72]. In particular, we observed very low genetic diversity within demes, and strong differences in allele frequencies (high *F_ST_* values) between demes (see text S1). Metapopulation dynamics involve frequent extinctions and recolonizations, thus creating strong genetic drift in local populations. In addition, selfing reduces the local effective population size and the frequency of gene exchange between individuals and populations, which reinforces the effects of genetic drift upon allelic frequencies [72], [73]. These results are consistent with previous population genetics and demographic studies conducted at more local scales on *Microbotryum* species infecting *S. latifolia* and *S. dioica*, also showing patterns consistent with metapopulation dynamics [42], [74]–[77].

### Genetic structure shaped by multiple glacial refugia

From a biogeographic point of view, Europe is a large peninsula with an East-West orientation, delimited in the south by a strong barrier, the Mediterranean Sea. During glaciation epochs, many species likely went through alternating contractions and expansions of range, involving extinctions of northern populations when the ice-sheet extended southward, and spread of the southern populations from different refugial areas as the glaciation receded. Such colonization processes were likely characterized by recurrent bottlenecks that would have led to lower diversity in the northern populations compared with the southern refugia [21]. The idea that refugia were localized in three areas (Iberia, Italy, Balkans) in Europe is now well-established [10], [11], [15], [17], although increasing evidence suggest that northern and eastern refugia also existed [78]–[84].

### *Microbotryum lychnidis-dioicae* (MvSl)

In MvSl, the strong phylogeographic structure observed at the European scale was composed of at least three genetic clusters with distributions strikingly similar to the major glacial refugia commonly recognized to have existed in Europe for many plant and animal temperate species (e.g., [14]). This pattern suggests that the pathogen likely colonized Northern Europe from at least the three main Mediterranean refugia (Iberian, Italian and Balkan).

The scenario may have been more complex, however, as the Eastern and Western clusters each further split into two groups, with divergence times of the five clusters roughly estimated between 200,000 and 350,000 yr BP. One of the eastern clusters was located north of the Balkans (mainly in Hungary and Czech Republic) and the other from Germany eastward. This pattern is consistent with colonization from distinct refugia located in the Balkans and further East in Eurasia, following a similar scenario as those reported in some animals, such as the bear *Ursus actor* [10], the vole *Myodes glareolus* [79], [80], some plant species [78], [85], [86], and also in some pathogens (e.g., [22]–[25]).

The two clusters identified in Western Europe had more diffuse geographic distributions, with a slight longitudinal partitioning. One of the clusters was distributed more towards the West of France while the second was more present in east-central France and in Eastern UK. Such a pattern may be due to the pathogen having survived in distinct regional refugia in Western Europe, from which they would have expanded their range over France and UK. Such a hypothesis is consistent with recent findings that the main glacial refugia in Europe were probably not composed of a single population, but instead could have been structured into several local refugia more or less isolated from one another (see the concept of “refugia within refugia” [87]–[91]).

While the north-south gradient in genetic diversity can be taken as a sign of range expansion from southern glacial refugia, a band of high genetic diversity was observed north of Italy and extending into Germany, as well as a hotspot of diversity in the centre of France. These areas of high of genetic diversity likely come from the colonization history of Europe by the different genetic clusters, establishing suture zones where genetic clusters meet and become intermingled. Such a pattern has been observed previously in a comparative approach of the history of colonization of 22 widespread and co-distributed European trees and shrubs [92], where hotspots of genetic diversity in the colonised ranges were found to be the result of mixed colonization from genetically isolated eastern and western European refugia [92]. The high genetic diversity found in MvSl in the Iberian peninsula also likely results from the co-occurrence of genotypes from four clusters.

Previous studies indicated that the phylogeographic pattern of the host plant *S. latifolia* similarly showed genetic evidence of post-glacial recolonization from Mediterranean refugia [56]. Analyses of cpDNA haplotypes revealed clear biogeographic structure in Europe, with haplotypes from Eastern and Western Europe forming divergent groups descending from haplotypes currently distributed in Iberian and Balkan Peninsulas [56]. The phylogeographic patterns in the plant *S. latifolia* and in its anther smut pathogen therefore seem to be congruent. In particular, the Eastern and Western clades identified in the host could correspond to the Eastern and Western genetic clusters in the pathogen MvSl. The pathogen however seems to display a finer genetic structure than its host, with particularly clear genetic evidence of an Italian glacial refugium for the pathogen, but not for the host plant (see figure 4 in [56]). More pronounced geographic structure is in fact expected in anther smut pathogens compared to their hosts, as has been observed at smaller scales [42]. This can be explained by the following observations: 1) the distribution of the pathogen is necessarily embedded within the range of its host, 2) anther smut pathogens are dispersed by the same vectors as the pollen of the plants, without being dispersed by seeds, so that their dispersal ability is lower than that of their host plants [42]. It is therefore likely that MvSl persisted in more fragmented refugia compared with its host. This highlights the potential use of pathogens as proxies for understanding host past migrations and distributions: the finding of distinct clusters in Italy and the Balkan in MvSl reveals that *S. latifolia* persisted in both these refugia during last glaciations, which was not obvious based solely on our current knowledge of the plant's phylogeograpy. However, statistically explicit comparative analyses linking the host and pathogen genetic polymorphisms, using comparable genetic markers, would be required to draw firm conclusions regarding correlations between the biogeographic structure of *S. latifolia* and MvSl (e.g. [93]).

### *Microbotryum silenes-dioicae* (MvSd)

The anther smut pathogen MvSd also exhibited a strong genetic structure, albeit with biogeographic patterns more difficult to interpret. The two main clusters had largely intermingled distributions, with an estimated time of divergence of the same order of magnitude as observed for MvSl. The distinct clusters in MvSd could correspond to genetic groups having diverged in distinct southern refugia during the glaciations, similar to MvSl, although the locations of the putative refugia are more difficult to identify. This may be due to the restricted distribution of MvSd, constrained by ecological specificities of the host and disease: the plant *S. dioica* is very rare in Mediterranean regions, and even more so the disease (we did not find anther smut symptoms on any *S. dioica* plant in the Pyrenees despite several years of searching). On the other hand, given the more northern current distribution of the plant *S. dioica* compared to *S. latifolia*, one can alternatively speculate that its tolerance to cold temperatures [62] might have allowed the host and the disease to remain in more northern refugia, as suggested for other species adapted to cold environments [84]. This could provide an explanation of the marginally significant increase in allelic richness with latitude in MvSd, although we cannot rule out that this pattern resulted from the co-occurrence of a greater number of different clusters in the north.

The phylogeography of the host plant *S. dioica* based on cpDNA RFLP similarly indicated the existence of genetically distinct groups, more or less longitudinally separated, albeit with large overlap in their ranges [58]. The distribution of the common cpDNA haplotypes was suggested to result from a post-glacial expansion of *S. dioica* across Europe from multiple southern refugia [57], [58]. However the absence of sampling from Mediterranean peninsulas in prior studies prevents any definitive conclusion regarding the number and location of these refugia. In addition, the geographic distribution of the shared haplotypes in *S. dioica* and *S. latifolia* was consistent with a history of hybridization and introgression events, making it difficult to assess whether the present distribution of these haplotypes resulted from the recolonization history of *S. dioica* or *S. latifolia* [58].

### Lack of gene flow among clusters and lack of IBD

A striking pattern observed in both *Microbotryum* species was the low level of admixture among genetic clusters (≤3%), suggesting almost complete lack of gene flow, despite the existence of contact zones. Such low levels of gene flow among clusters are likely influenced by the very high selfing rates in *Microbotryum*. High selfing rates have been invoked to explain reproductive isolation between sympatric *Microbotryum* species [67], [94], and there could be a similar effect in keeping the genetic clusters distinct within species. The high selfing rates also explain why increasing the number of clusters in Bayesian analyses always increased the explanatory value in describing the population genetic structure, without the appearance of admixed clusters, even for very high K values: this is because each diploid individual mostly reproduces with itself and therefore the smallest ‘panmictic unit’ may indeed be the individual. In selfing species, the genetic structure extends to a much finer scale than in outcrossing species [32], [95]. The lack of gene flow among clusters may result in addition to metapopulation dynamics and rapid expansion during post-glacial recolonization. A theoretical study [96] indeed showed that rapid growth in population size after founding events resulted in gene frequency divergence that is resistant to decay by gene exchange.

### Inference for climate change and emerging infectious diseases

Large-scale congruence between the pathogens' phylogeographic patterns and those of their respective hosts indicates that their glacial refugia and migration pathways during recolonization have been similar. While this may be expected for obligate pathogens like *Microbotryum* species, highly dependent on their hosts for survival and using the same dispersal vectors, we interestingly found that the pathogens likely subsisted during glaciations in a more fragmented distribution, with their genetic diversity divided among a higher number of smaller refugia. Moreover, the extent of large-scale dispersal across Europe after recolonization was less for the pathogen than for its host: in particular, the clusters were much more clumped in MvSl than in *S. latifolia*, and footprints of refugia appeared in MvSl that were absent in *S. latifolia*, such as the Italian peninsula. Our findings thus indicate that vector-borne, obligate pathogens may colonize new areas following climate warming with some delay compared to their hosts, and to a lesser extent. The invasive potential of pathogens following climate change is therefore likely to depend on the obligate nature of the interactions with their host and on the dispersal modes, as could be expected.

However, once the original host and its fungal pathogen invade a geographic region, the pathogen poses a risk of emerging as an infectious disease on new host species found in that area. For instance, MvSl was introduced in the United States some time after its host plant *S. latifolia*, and has remained in a much more restricted geographic area [97]. Cross-species disease transmission was nevertheless documented in the United States to another non-native species, *S. vulgaris*, that is otherwise free of anther smut disease in the continent [98]. In Europe, we have previously detected rare events of cross-species disease transmission between *S. dioica* and *S. latifolia* and of hybridization between MvSl and MvSd that occurred after secondary contact [52]. Host shifts are frequent in fungal pathogens [4], [99], [100] in particular in *Microbotryum*, where co-phylogenetic analyses showed that speciation events were most often associated with host shifts [47]. This suggests that climate warming may cause emerging infectious diseases, by resulting in contacts between different potential hosts that were allopatric, even when the intrinsic dispersal capacity of the pathogens is limited and their migration pathways are constrained by those of their hosts. Climate warming can also bring into contact differentiated populations from the same species, promoting introgression between previously geographically isolated populations, which can have important and unpredictable evolutionary consequences. We showed in the present study that secondary contact between genetically differentiated clusters happened after the glaciations in MvSl, and that the highly selfing mating system was here important in preventing introgression.

The substantive contrast in phylogeography for the anther smut fungi on *S. latifolia* and *S. dioica*, which may be attributed to differences in the hosts' ecology, is also relevant for predicting the fate of infectious diseases following global warming. The redistribution of pathogens under warmer climatic conditions should indeed be highly dependent on the hosts' ecological preferences and adaptive potentials, in particular regarding the temperature and competition in new ecological communities [39]–[41].

### Conclusion

In this study, we showed that high selfing rates and metapopulation dynamics in two plant pathogenic fungi had strong impact on their genetic diversity and structure. At the scale of the species' distribution ranges, the population structures in the two fungal species were quite different, likely due to differences in the ecological preferences of the two host-pathogen systems. The broadly distributed *S. latifolia* and its anther smut pathogen have kept clear genetic footprints of postglacial colonization from the major southern European refugia. The pathogens showed striking evidence for more numerous and localized refugia than their hosts. On the other hand, the ecological preference of the plant *S. dioica* for wetter and colder habitats [62] probably led to a more restricted and more northern distribution of the plant and its anther smut pathogen, and may have induced a drastic contraction of their distribution ranges with the post-glacial warming and the fragmentation of suitable habitat conditions. The European genetic structures of the anther smut fungi seem to match those of their respective hosts, with even a finer genetic structure, so that the geographic distribution of genetic variation in the pathogens may be useful to draw inferences about host phylogeography.

Beyond the interest of our study for understanding the dynamics of diseases under climate warming and the impact of host life histories on the genetic structure of pathogens, our study illustrates several important points to take into account when performing clustering genetic analyses, which are still often poorly recognized. First, several K values are often interesting to consider in clustering analyses, and it may be non-heuristic to search for a “single optimal” number of clusters. As long as increasing K does not lead to admixed clusters, the new clusters revealed by increasing K probably reveal a genuine genetic structure that may be interesting to investigate. This appears especially true in selfing species, for which the smallest panmictic cluster may be the individual.

## Materials and Methods

### Teliospore collection and populations

The individuals of *Microbotryum* analyzed in this study were collected as diploid teliospores from 187 localities on *S. latifolia* (n = 701) and 68 localities on *S. dioica* (n = 342) across Europe (Figure S10) and stored in silica gel (see Figure S1 for a detailed description of the sampling). DNA from teliospores of one flower per diseased plant was extracted for genetic analyses. Multiple infections by different genotypes are frequent in the *Silene*-*Microbotryum* system, but teliospores within a single flower originate from a single diploid individual [101].

### Microsatellite genotyping

DNA was extracted as described in [77]. Teliospores were genotyped using 11 microsatellite markers following the protocol of [77] (Table 1). Among the 11 microsatellite loci used, E14, E17, E18 were described in [102], SL8, SL9, SL12, SL19, SVG5, SVG8, SVG14 described in [103], and SL5 was described in [104].

### Data analyses

#### Sample handling

Hybridization between MvSl and MvSd has been suggested previously [105]. However, we showed recently in a study investigating the divergence process between the two species using the same dataset as in the present work that hybridization was very rare in natural populations [52]. Therefore, we investigated here the genetic polymorphism and population structure in MvSl and MvSd separately, after having removed the few inter-specific hybrids and cross-disease species transmissions from the datasets (see [52] for more details).

#### Descriptive statistics

The within-species genetic polymorphism at each locus was quantified using the allelic richness (*A_r_*), the observed and unbiased expected heterozygosities (*H_o_* and *H_e_*), and the fixation index (*F_IS_*). These statistics were calculated using FSTAT 2.9.3.2 [106]. Departure from Hardy-Weinberg expectations was tested using exact tests implemented in GENEPOP 4.0 [107], [108].

Linkage disequilibrium (LD) among loci was quantified using the correlation coefficient (*r^2^*) calculated in Genetix 4.05 [109]. In order to assess the impact of mating system on the genetic structure, we calculated *r^2^* within sampled localities that contained at least 10 genotypes, i.e. 15 out of 187 localities for MvSl (n = 210, Figure S11) and 13 out of 68 localities for MvSd (n = 197, Figure S12). Genotypic disequilibrium was tested using permutation test implemented in FSTAT 2.9.3.2. The nominal *P-value* of 0.05 was adjusted for multiple comparisons using a Bonferroni correction (i.e. 6.1×10^−5^, based on 82,500 permutations).

#### Population structure

We investigated population structure in MvSl and MvSd using two kinds of analyses: Bayesian model-based clustering and principal component analyses. We applied three variants of Bayesian model-based clustering algorithms. The first one, implemented in STRUCTURE 2.3 [110], [111], partitions multilocus genotypes into clusters while minimizing departure from Hardy Weinberg and linkage equilibrium (HWLE) among loci [110], [111]. The second one, implemented in the InStruct software, is a variant of the previous one specifically suited to selfing organisms [112], such as *Microbotryum* species (e.g. [69]–[71], [77]). InStruct relaxes the assumption of Hardy-Weinberg equilibrium through the calculation of the expected genotype frequencies on the basis of selfing rates (jointly inferred from the data) rather than as simple products of allele frequencies [112]. The third one implemented in TESS 2.3.1 [64], [113], [114] differs from the STRUCTURE algorithm in including spatially explicit prior distributions describing which sets of individuals are likely to have similar cluster membership [113]. In this approach, clusters correspond to spatially and genetically continuous units separated by small discontinuities that occur where genetic barriers are crossed. The incorporation of a spatial component into the clustering model has the potential to determine if clines provide a sensible description of the underlying pattern of variation [64].

For both STRUCTURE and TESS algorithms, we used a haploid setting because both species of *Microbotryum* are almost completely homozygous (Table 1). Run conditions for STRUCTURE analyses were as follows: we conducted a series of independent runs with different proposals for the number of clusters (K), testing all values from 1 to 15. Each run used 500,000 iterations after a burn-in of 250,000 iterations, using a model allowing for admixture and correlated allele frequencies. To ensure convergence of the MCMC, we performed 10 independent replicates for each value of K and checked the consistency of results visually and using the procedure implemented in the program CLUMPP 1.1.1 [115]. We used CLUMPP 1.1.1 to account for label switching. We also used CLUMPP 1.1.1 to compute with the *Greedy* algorithm the symmetric similarity coefficient between pairs of runs (100 random input sequences, G' statistic), in order to identify potential distinct modes among the results of independent replicate runs for each K value and to average individual assignment probabilities (*q*) over replicated runs showing a similar mode. Barplots were generated by the DISTRUCT program [116]. We attempted to identify the number(s) of clusters (K) that best explained the data using the probability of the data under the considered value of K, i.e. Ln(Pr(X|K)) where X are the data (Ln(D) in [117]) and its rate of change when increasing K.

InStruct run conditions were as follows: we used diploid data to jointly estimate the selfing rate within each clusters and the individual assignment probability (*q*) to each cluster [112]. We ran 10 independent replicates for each K, testing all values between 1 and 10. Each MCMC chain used 1,000,000 iterations after a burn-in of 200,000 iterations and a thinning interval of 50. The convergence of each run was tested using CLUMPP in a similar way to that described above for the STRUCTURE analysis, and barplots were produced using DISTRUCT. InStruct does not estimate Ln(Pr(X|K)), but it computes instead a Deviation Index Criterion (DIC), i.e. a model-complexity penalized measure of how well the model fits the data [118].

TESS run conditions were as follows: we used the conditional auto-regressive (CAR) Gaussian model of admixture with linear trend surface [64], and set the admixture parameter to α = 1 and the interaction parameter *ρ* = 0.6 as starting values and subsequently updated. The algorithm was run with a burn-in period of length 20,000 cycles, and estimation was performed using 30,000 additional cycles. We increased the maximal number of clusters from K_max_ = 2 to K_max_ = 15 (10 replicates for each value). For K_max_ = 5, we performed 50 additional longer runs (burn-in 30×10^3^, run-length 100×10^3^, ρ = 0.6, α = 1), and we averaged the estimated admixture coefficients (*Q* matrix) over the 5 runs with the smallest values of the DIC (DIC  = 14 819, s.d. = 9). To account for label switching among runs, we used the software CLUMPP version 1.1, whose greedy algorithm computed a symmetric similarity coefficient equal to 0.85 (100 random input sequences, G' statistic).

Finally, we applied a centred-normed Principal Component Analysis (PCA) on the microsatellite allele frequencies for each species to corroborate the clustering solution inferred by the three Bayesian clustering algorithms using a multivariate approach that does not rely on any model assumption. We conducted the analyses using ADEgenet [119] and ADE4 packages [120] in the R-environment [121].

We represented the relationships among clusters using neighbour-joining population trees, respectively based on Nei's *D_A_* distance [122], shared allele distance *D_SA_* [123], [124], Chord's distance [125] and Goldstein's (*δµ*)^2^ distance, the latter one assuming that microsatellite evolve following a Stepwise Mutation Model (SMM) model [126]. The computation of distance matrix and of bootstrapped distances was performed using *PowerMarker* [127], and the consensus tree was obtained and plotted using MEGA 4 [128]. As the different trees provided similar topologies, only the tree based on the D_a_ distance of Nei et al. [122] is presented. This distance was found to perform comparatively well in estimation of population trees from microsatellite allele frequency data [129]. The root was placed between the two species.

#### Spatial pattern in genetic diversity

Allelic richness and private allelic richness of each genetic cluster or combination of clusters identified in the Bayesian clustering analysis were calculating using ADZE 1.0 [130]. The expected heterozygosity (He) was calculated using FSTAT 2.9.3. Variation in allelic richness across space was analysed by aggregating samples according to a grid system whose mesh size was chosen in order to have at least 4 samples per cell (132×139 km). This cut-off value was chosen as a trade-off between the sample size per cell and its spatial distribution. This led for MvSl to 66 cells, out of which 47 contained at least 4 samples, and for MvSd to 31 cells, out of which 19 had at least 4 samples. Spatial interpolation was performed using a thin plate spline method, with a smoothing parameter of λ = 0.005, as implemented in the R package ‘fields’ [121].

#### Isolation by distance (IBD) analyses

We used pairwise kinship coefficients between individuals (*F_ij_*) (Loiselle *et al.* 1995) to test for isolation by distance, as recommended for highly selfing species [65]. We calculated the average kinship coefficient for different intervals of distance ranges (10, 100, 500, 1500 and 5000 km), the slope of the correlation coefficient between individual kinship coefficients and the logarithm of geographic distance, and the significance of the slope (10,000 permutations of localities) using the software SPAGeDi 1.3 [131]. When the slope was significant, we calculated the ‘Sp’ statistic, which is the ratio
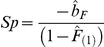
where 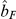 is the slope of the correlation coefficient and 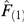 is the mean *F_ij_* between individuals belonging to a first distance interval that includes all pairs of neighbours (in our case, this distance interval corresponds to a local population). Under some assumptions, the Sp statistic is equal to the inverse of 4π*Dσ* ^2^ (with *D*: effective population density and *σ* ^2^: average squared axial parent-offspring distance), which matches Wright's neighbourhood size, under the hypothesis of Gaussian dispersal functions. Therefore, high values of the Sp statistic are indicative of low population density or limited dispersal ability.

## Supporting Information

Figure S1 Map of sampled localities for *Microbotryum lychnidis-dioicae* (MvSl, n = 701) and *M. silenes-dioicae* (MvSd, n = 342).
(0.68 MB TIF)

Figure S2 Estimated number of populations in *Microbotryum lychnidis-dioicae* (MvSl) from STRUCTURE (a and b), InStruct (c) and TESS (d) analyses. STRUCTURE analyses: (a) mean (± SD) probabilities of the data [LnPr(X|K)] over 10 Structure replicated runs plotted as a function of the putative number of clusters (K). (b) Mean variations of probabilities of the data (Δ(LnPr(X|K)) between successive K considered in STRUCTURE analyses. Deviation Index Criterion (DIC) for InStruct (c) and TESS (d) analyses.
(0.16 MB TIF)

Figure S3 Estimated population structure from the STRUCTURE, InStruct and TESS analyses assuming 5 clusters. Each individual is represented by a thin horizontal line divided into K coloured segments that represent the individual's estimated membership fractions in K clusters. Black lines separate individuals from different geographic areas labelled on the right. Each plot is based on the dominant clustering solution identified at that value of K.
(0.46 MB TIF)

Figure S4 Population structure inferred from InStruct analyses assuming 2 to 8 clusters. Each individual is represented by a thin vertical line divided into K coloured segments that represent the individual's estimated membership fractions in K clusters. Black lines separate individuals from different geographic areas. Several barplots are shown for each K and represent the distinct modal solutions observed.
(5.37 MB TIF)

Figure S5 Principal component analysis on microsatellite allelic frequencies of *Microbotryum lychnidis-dioicae* (MvSl). Scatter plots for the first four principal components are shown using a colour labelling of genotypes defined according to the membership probability to belong to the 5 identified clusters using Bayesian clustering analyses. Each genotype that received a probability above 0.7 was coloured according to the colour pattern used in Bayesian clustering, otherwise it was considered as admixed and coloured in orange.
(0.33 MB TIF)

Figure S6 Principal component analysis on microsatellite allelic frequencies of *Microbotryum lychnidis-dioicae* (MvSl). Maps for the first four principal components scores (PC1 to 4) are shown.
(0.97 MB TIF)

Figure S7 Same as Figure S2, but for *Microbotryum silenes-dioicae* (MvSd).
(0.15 MB TIF)

Figure S8 Same as Figure S4, but for *Microbotryum silenes-dioicae* (MvSd).
(0.76 MB TIF)

Figure S9 Principal component analysis on microsatellite allelic frequencies of *Microbotryum silenes-dioicae* (MvSd) for K = 2, 3 and 5. Scatter plots for the first four principal components are shown using a colour labelling of genotypes defined according to the membership probability to belong to the K identified clusters using Bayesian clustering analyses. Each genotype that received a probability above 0.7 was coloured according to the colour pattern used in Bayesian clustering, otherwise it was considered as admixed and coloured in orange.
(0.21 MB TIF)

Figure S10 Level of inter-sites differentiation, expressed a FST value, as a fonction of their geographic distance in *Microbotryum silenes-dioicae* (MvSd).
(0.05 MB TIF)

Figure S11 Mapped values overall loci for several descriptive statistics in *Microbotryum lychnidis-dioicae* (MvSl) population where the sample size was at least of 10 teliospores. The statistics shown include the sample size per locations (N), the mean allelic richness (Ar) per population, the mean observed and expected heterozygosity (Ho and He), the average number of fixed loci per populations (Nb. fixed loci), the overall loci inbreeding coefficient (FIS) per population, and the linkage disequilibrium across loci estimated using the correlation coefficient (*r^2^*) averaged across loci. We used the function quilt.plot in the R “Fields” package to plot the values on a map according to a grid system of 50×33 pixels. Locations that fall into the same grid box will have their values averaged.
(0.75 MB TIF)

Figure S12 Same as Figure S11, but for *Microbotryum silenes-dioicae* (MvSd). The locus SL19 was excluded in the calculation of Ho, He, FIS, to avoid bias related to its almost complete fixation in a heterozygous state (see the text).
(0.58 MB TIF)

Text S1 Description of genetic polymorphism in *Microbotryum lychnidis-dioicae* (MvSl) and *M. silenes-dioicae* (MvSd).
(0.07 MB PDF)

Table S1 Level of pairwise differentiation, estimated as F_ST_ value, among samples of *M. lychnidis-dioicae* where n was higher than 10 strains. All pairwise values were significantly different at p<0.0001.
(0.05 MB PDF)

Table S2 Same as Table S1 but for *Microbotryum silenes-dioicae* (MvSd) populations. All pairwise values but one (*) were significantly different at p<0.0001.
(0.05 MB PDF)
