# Supplementary figures and images for: Glacial Refugia in Pathogens: European Genetic Structure of Anther Smut Pathogens on Silene latifolia and Silene dioica
Source: PLoS Pathog. 2010 Dec 16;6(12):e1001229. doi: 10.1371/journal.ppat.1001229 (PMC3002987; doi:10.1371/journal.ppat.1001229)

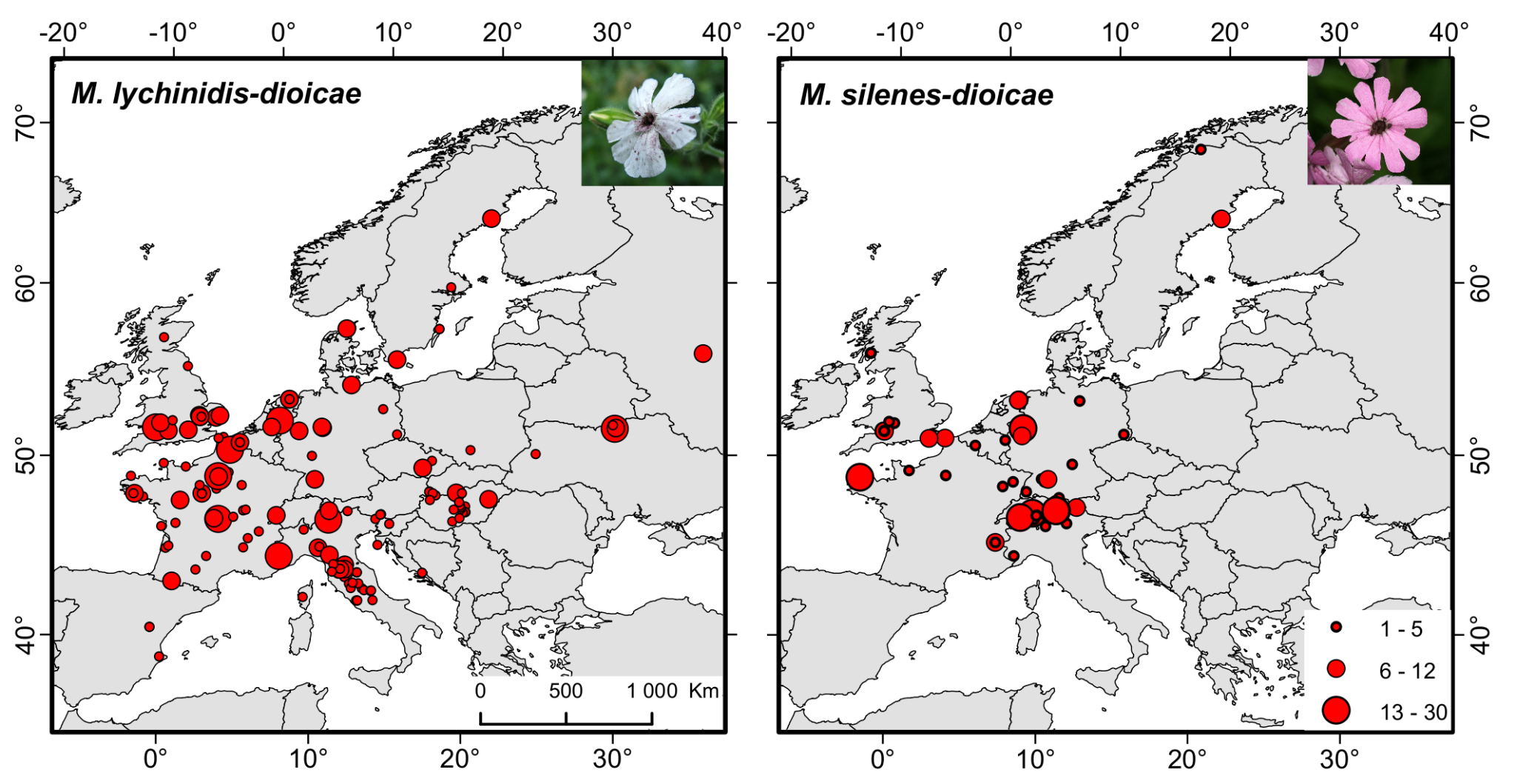

Supplement: Figure S1 — Map of sampled localities for Microbotryum lychnidis-dioicae (MvSl, n = 701) and M. silenes-dioicae (MvSd, n = 342). (0.68 MB TIF) [file ppat.1001229.s001.tif]

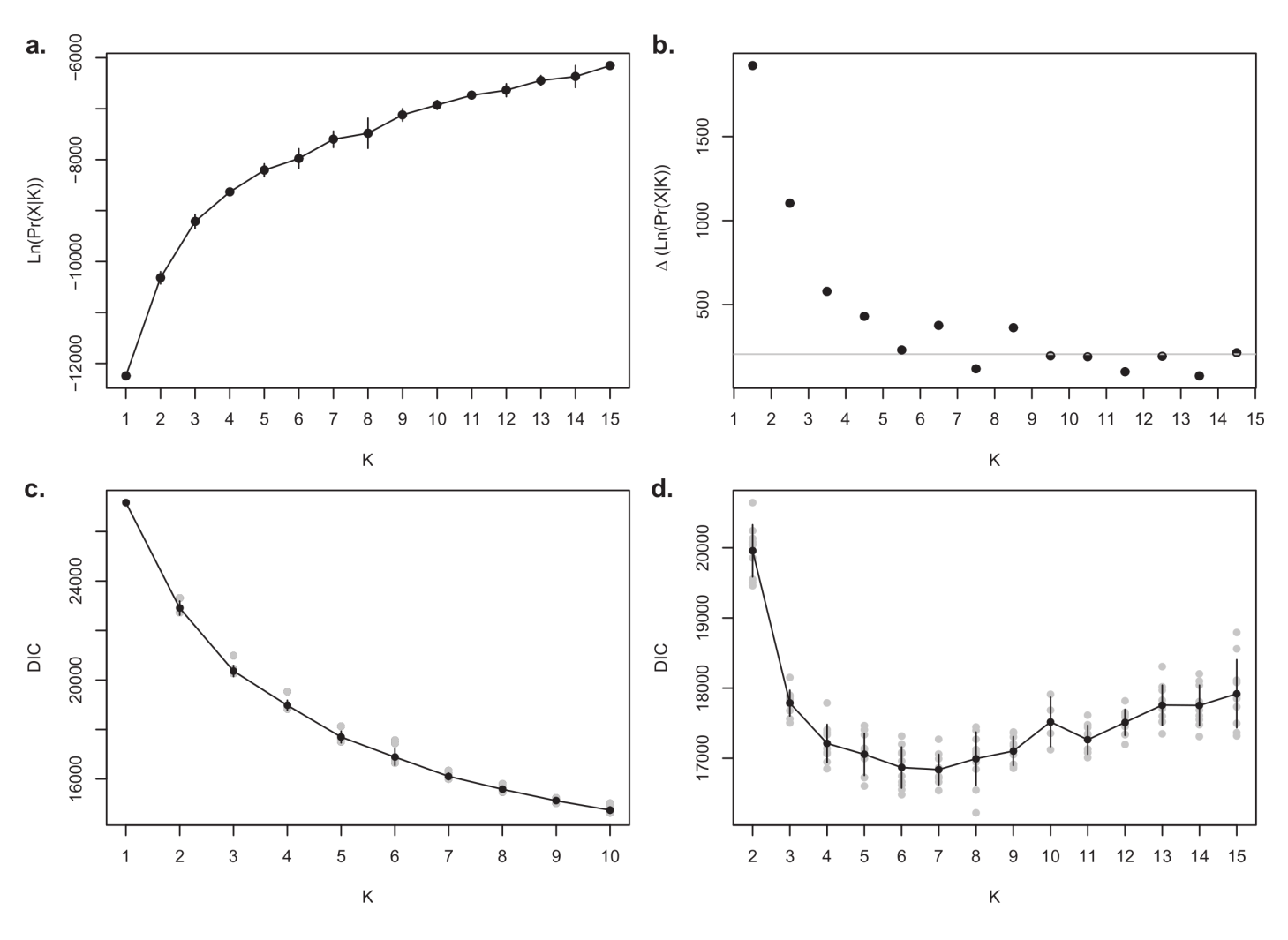

Supplement: Figure S2 — Estimated number of populations in Microbotryum lychnidis-dioicae (MvSl) from STRUCTURE (a and b), InStruct (c) and TESS (d) analyses. STRUCTURE analyses: (a) mean (± SD) probabilities of the data [LnPr(X|K)] over 10 Structure replicated runs plotted as a function of the putative number of clusters (K). (b) Mean variations of probabilities of the data (Δ(LnPr(X|K)) between successive K considered in STRUCTURE analyses. Deviation Index Criterion (DIC) for InStruct (c) and TESS (d) analyses. (0.16 MB TIF) [file ppat.1001229.s002.tif]

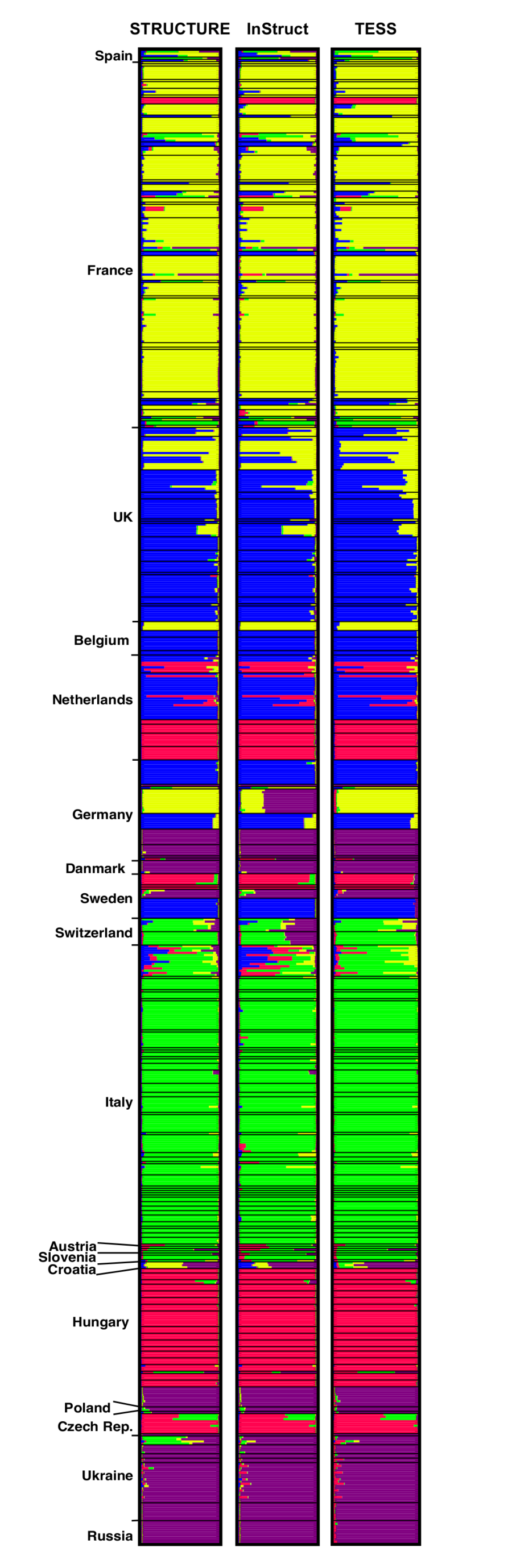

Supplement: Figure S3 — Estimated population structure from the STRUCTURE, InStruct and TESS analyses assuming 5 clusters. Each individual is represented by a thin horizontal line divided into K coloured segments that represent the individual's estimated membership fractions in K clusters. Black lines separate individuals from different geographic areas labelled on the right. Each plot is based on the dominant clustering solution identified at that value of K. (0.46 MB TIF) [file ppat.1001229.s003.tif]

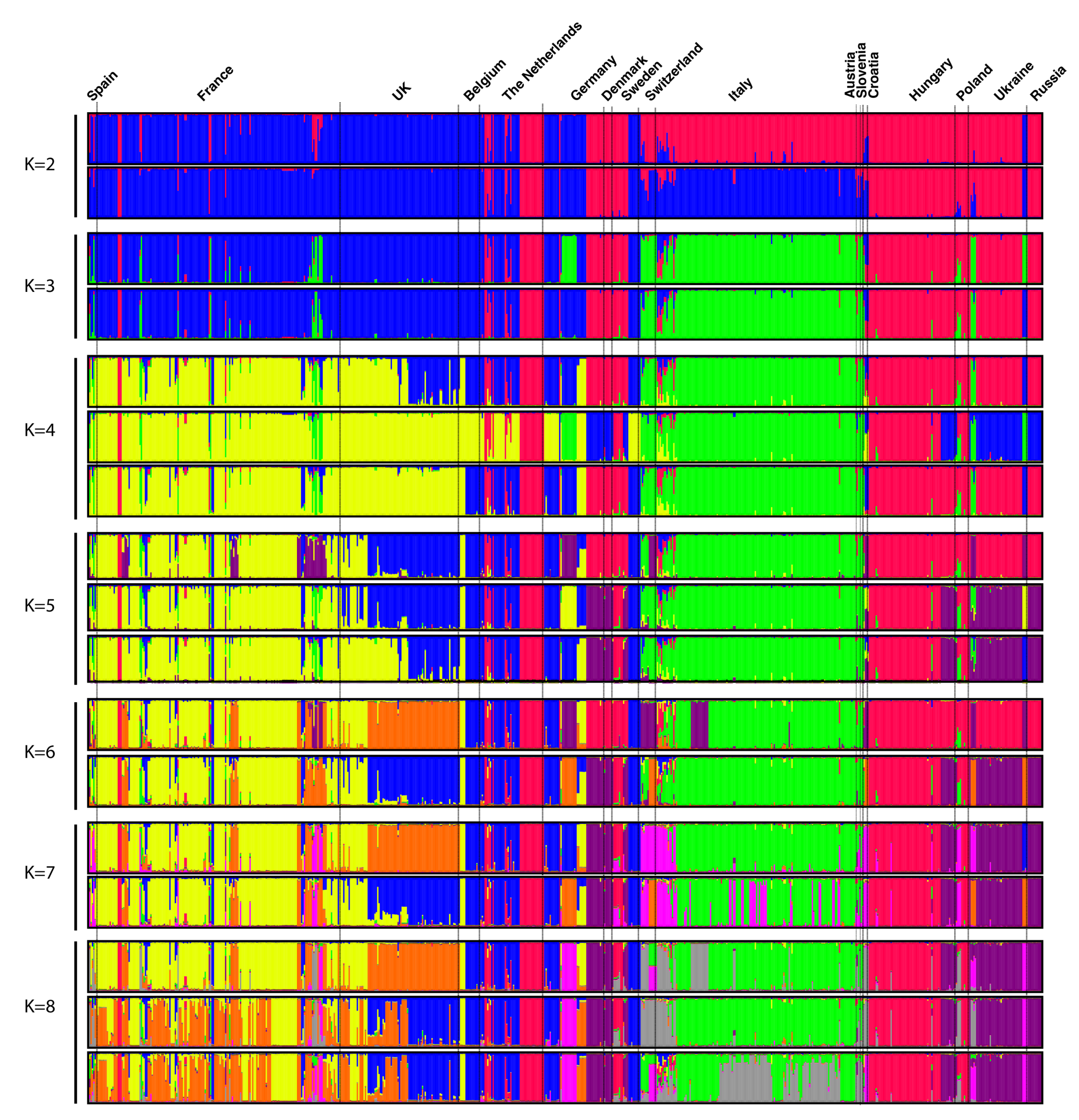

Supplement: Figure S4 — Population structure inferred from InStruct analyses assuming 2 to 8 clusters. Each individual is represented by a thin vertical line divided into K coloured segments that represent the individual's estimated membership fractions in K clusters. Black lines separate individuals from different geographic areas. Several barplots are shown for each K and represent the distinct modal solutions observed. (5.37 MB TIF) [file ppat.1001229.s004.tif]

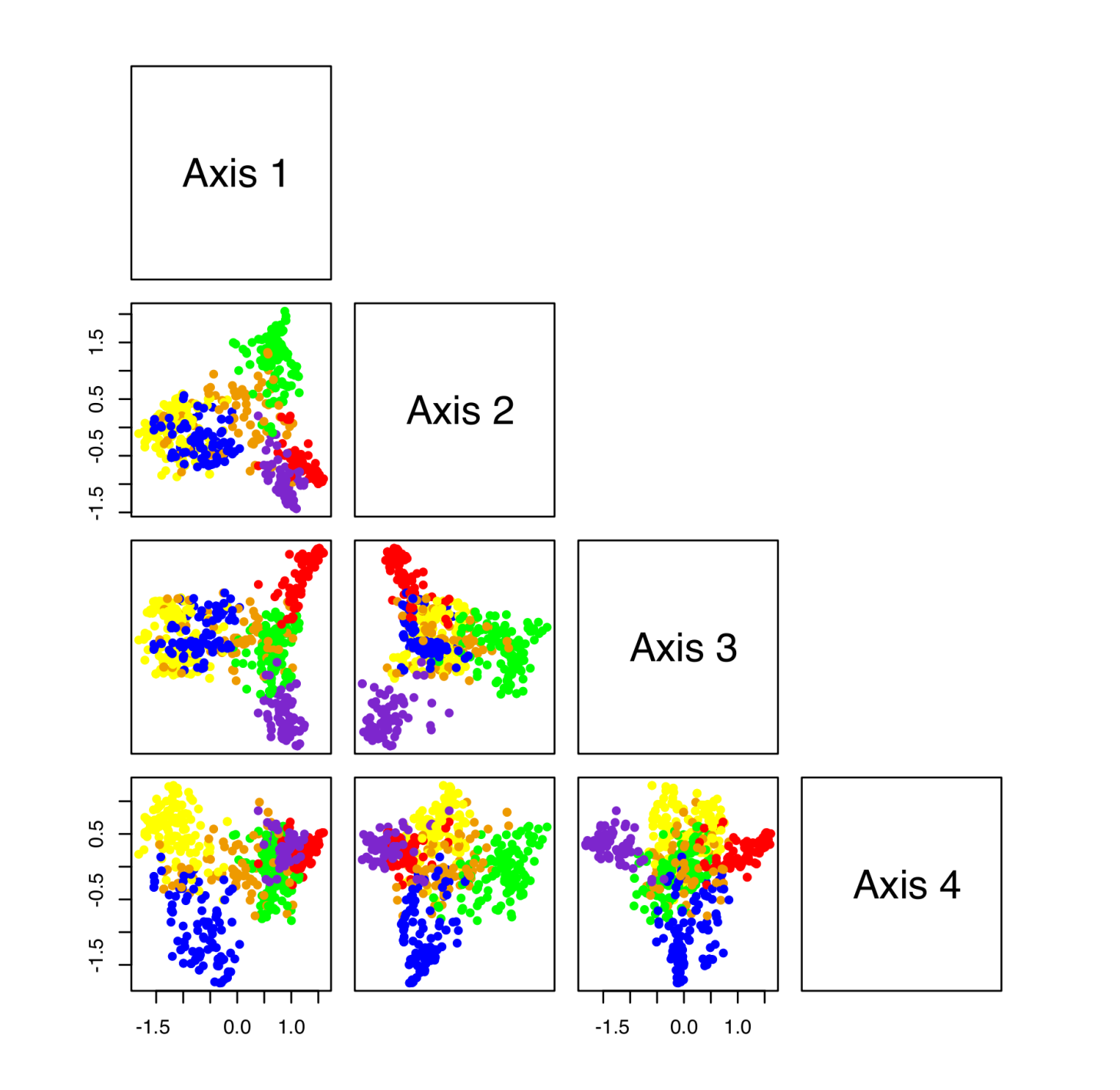

Supplement: Figure S5 — Principal component analysis on microsatellite allelic frequencies of Microbotryum lychnidis-dioicae (MvSl). Scatter plots for the first four principal components are shown using a colour labelling of genotypes defined according to the membership probability to belong to the 5 identified clusters using Bayesian clustering analyses. Each genotype that received a probability above 0.7 was coloured according to the colour pattern used in Bayesian clustering, otherwise it was considered as admixed and coloured in orange. (0.33 MB TIF) [file ppat.1001229.s005.tif]

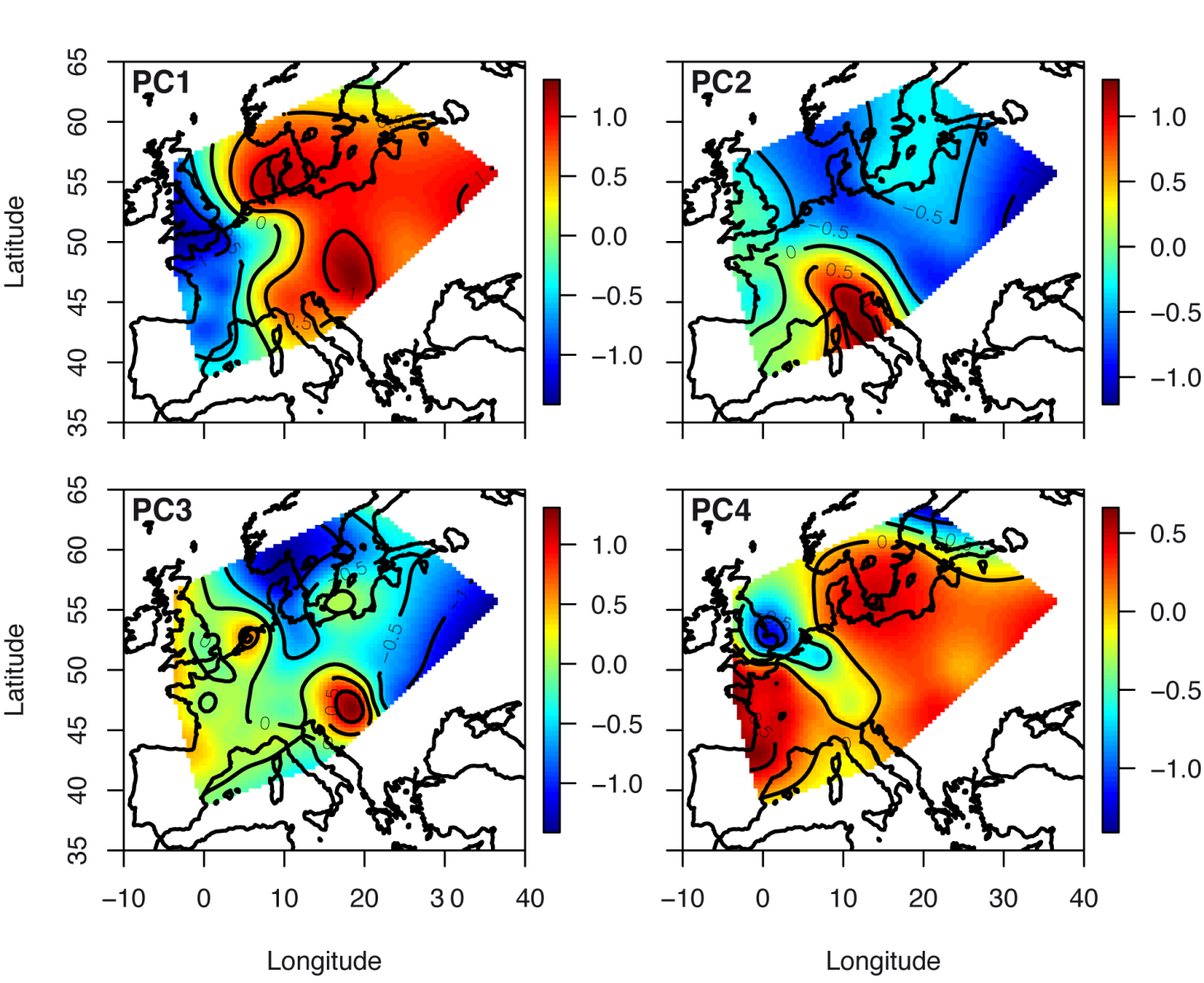

Supplement: Figure S6 — Principal component analysis on microsatellite allelic frequencies of Microbotryum lychnidis-dioicae (MvSl). Maps for the first four principal components scores (PC1 to 4) are shown. (0.97 MB TIF) [file ppat.1001229.s006.tif]

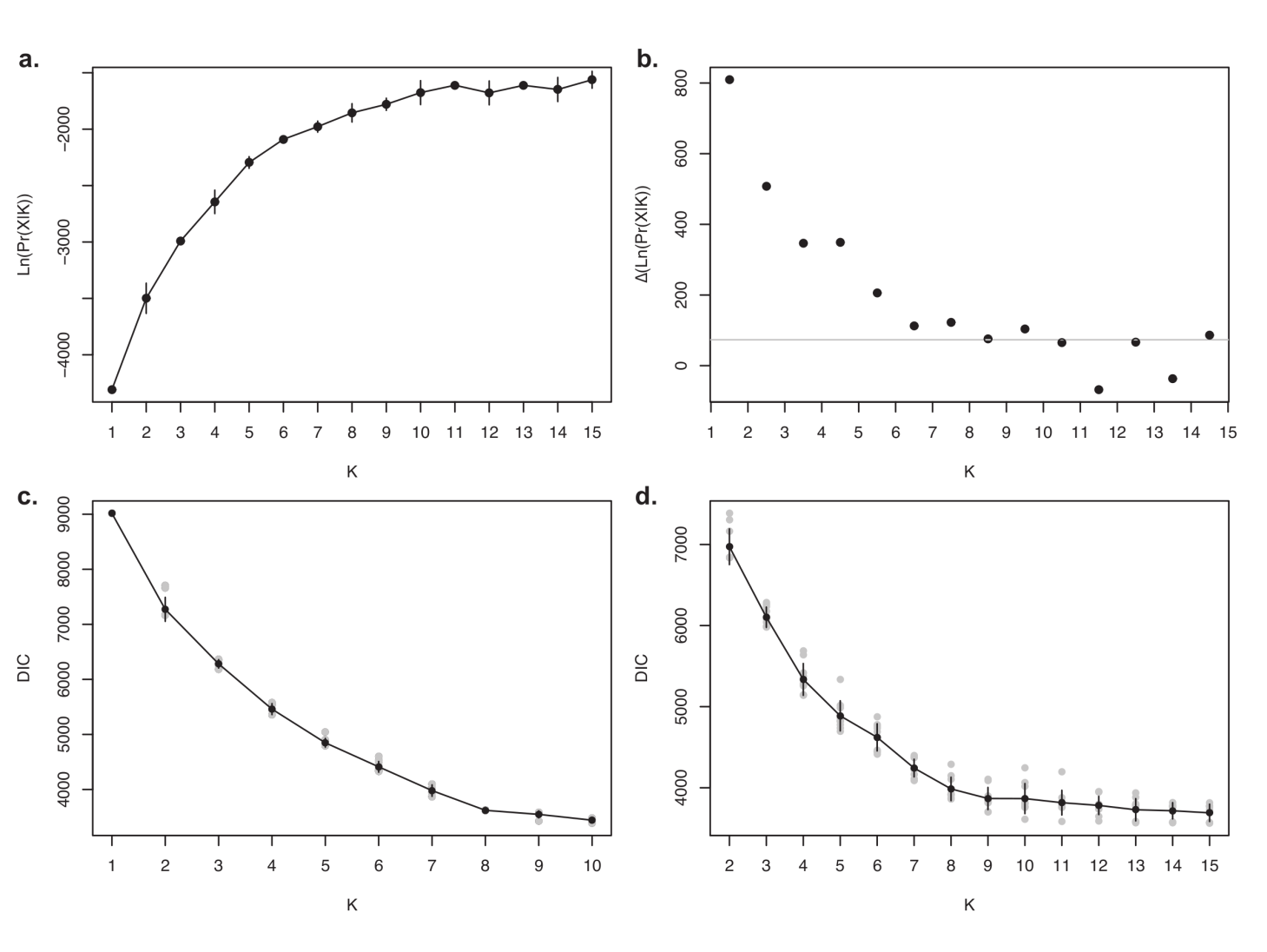

Supplement: Figure S7 — Same as Figure S2, but for Microbotryum silenes-dioicae (MvSd). (0.15 MB TIF) [file ppat.1001229.s007.tif]

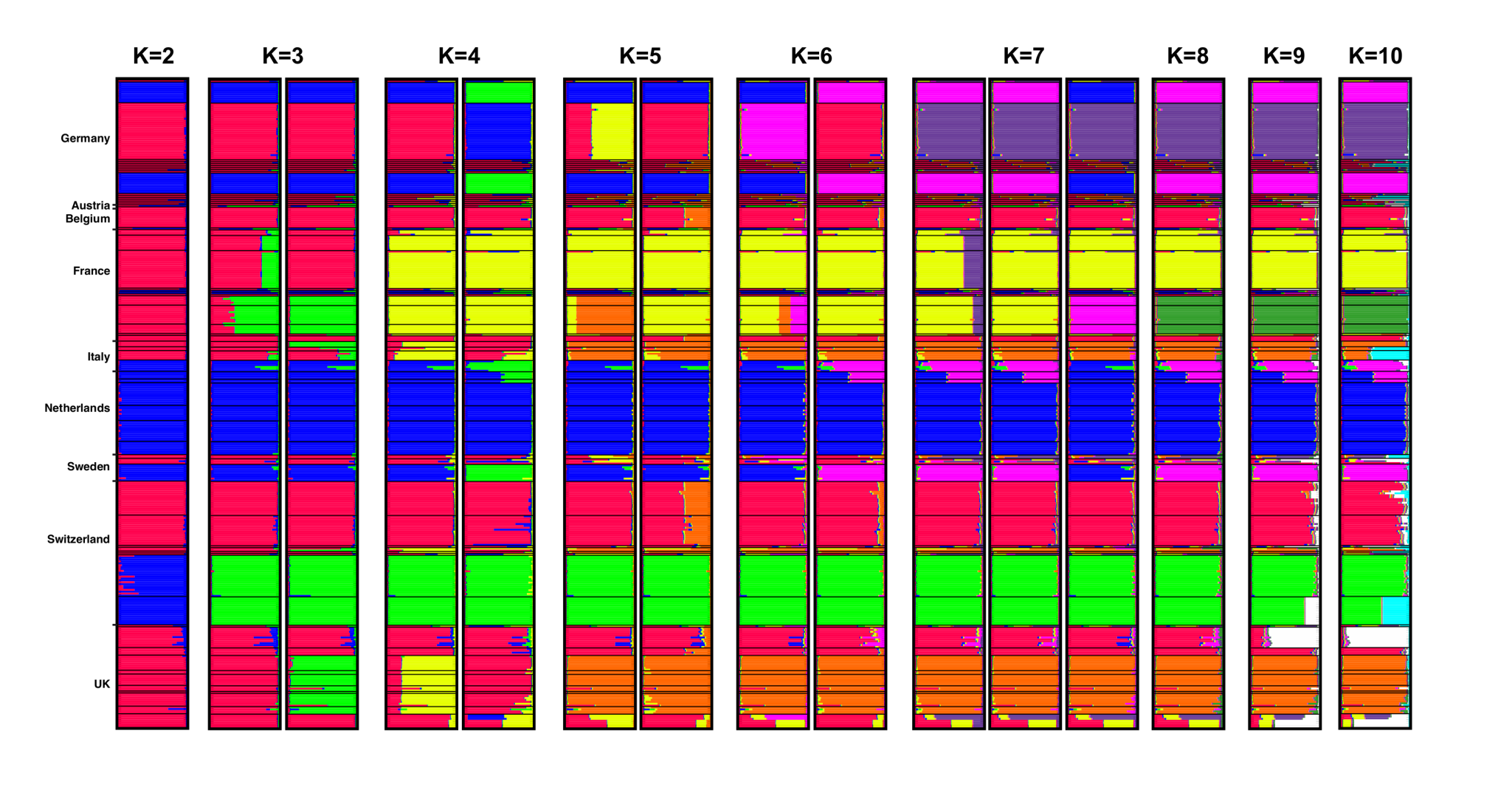

Supplement: Figure S8 — Same as Figure S4, but for Microbotryum silenes-dioicae (MvSd). (0.76 MB TIF) [file ppat.1001229.s008.tif]

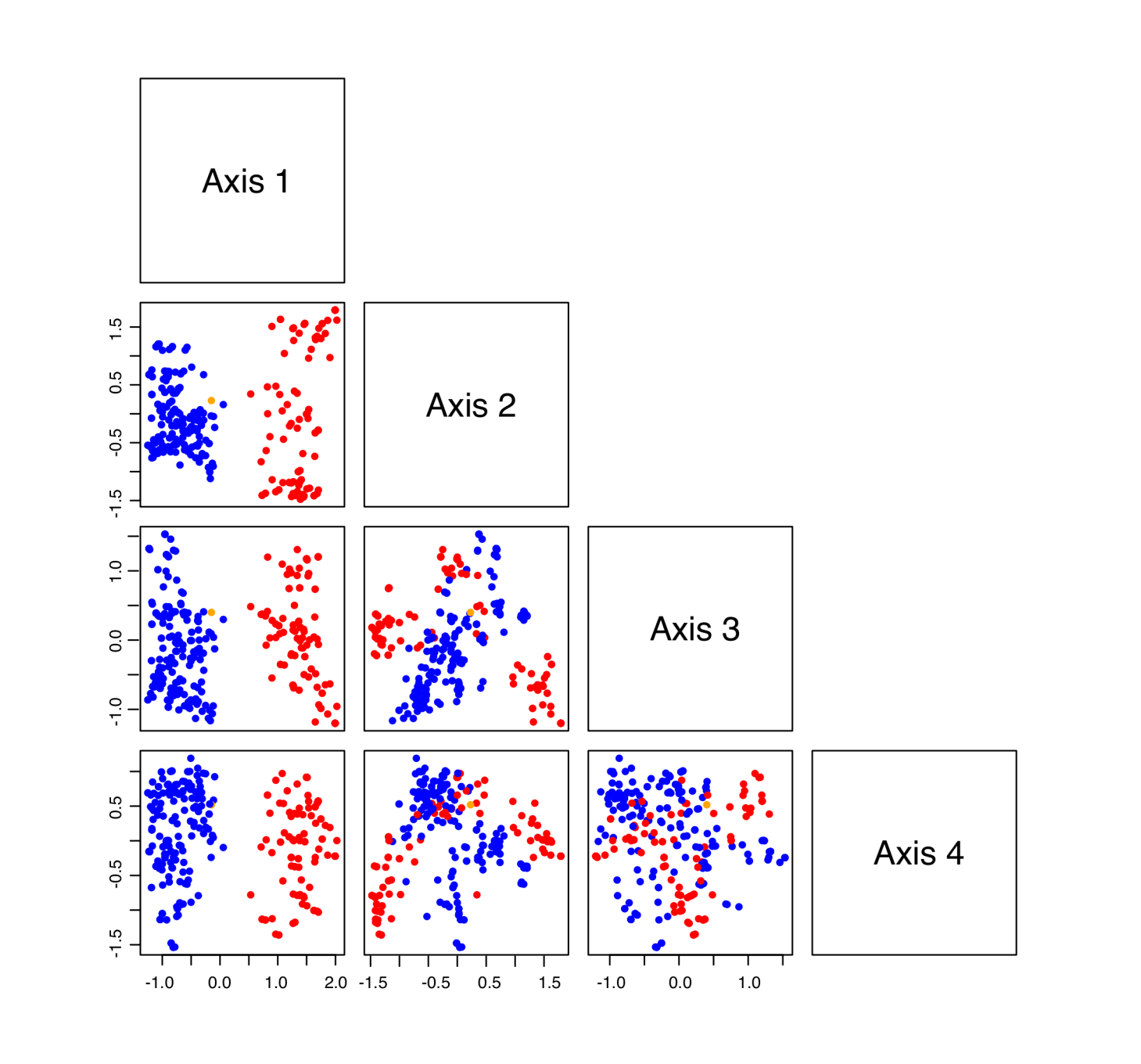

Supplement: Figure S9 — Principal component analysis on microsatellite allelic frequencies of Microbotryum silenes-dioicae (MvSd) for K = 2, 3 and 5. Scatter plots for the first four principal components are shown using a colour labelling of genotypes defined according to the membership probability to belong to the K identified clusters using Bayesian clustering analyses. Each genotype that received a probability above 0.7 was coloured according to the colour pattern used in Bayesian clustering, otherwise it was considered as admixed and coloured in orange. (0.21 MB TIF) [file ppat.1001229.s009.tif]

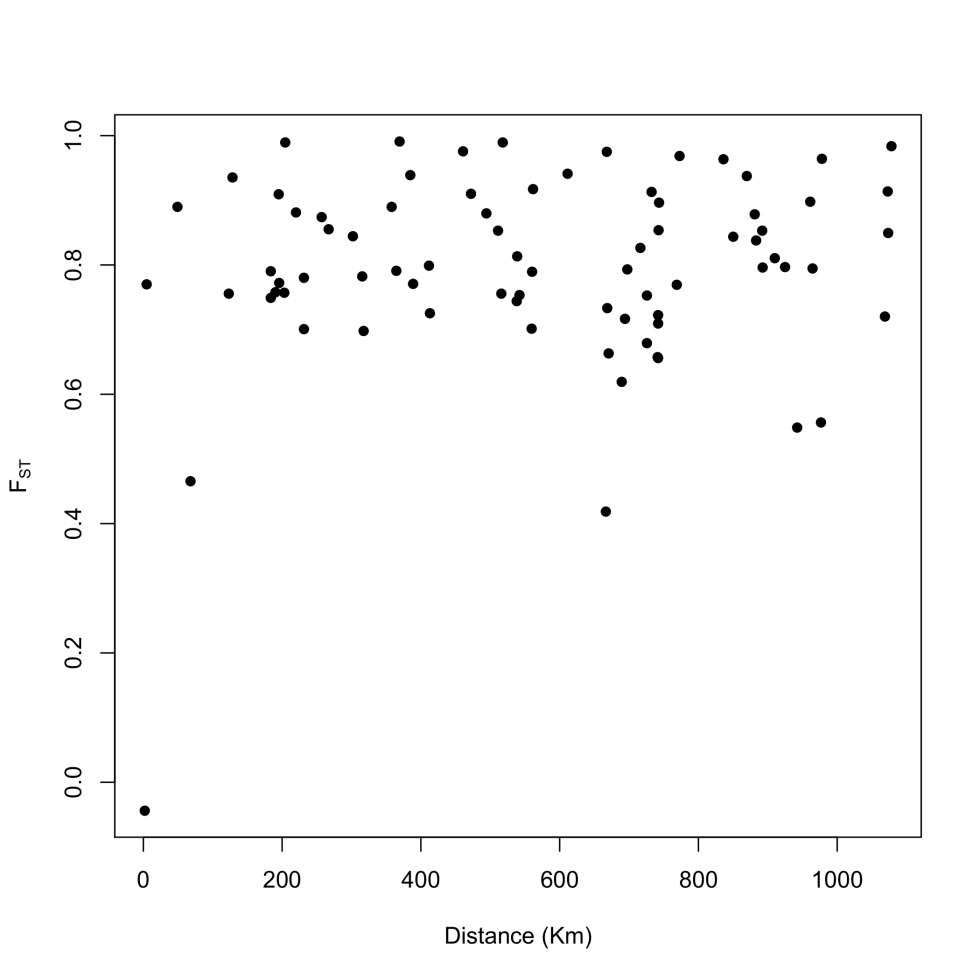

Supplement: Figure S10 — Level of inter-sites differentiation, expressed a FST value, as a fonction of their geographic distance in Microbotryum silenes-dioicae (MvSd). (0.05 MB TIF) [file ppat.1001229.s010.tif]

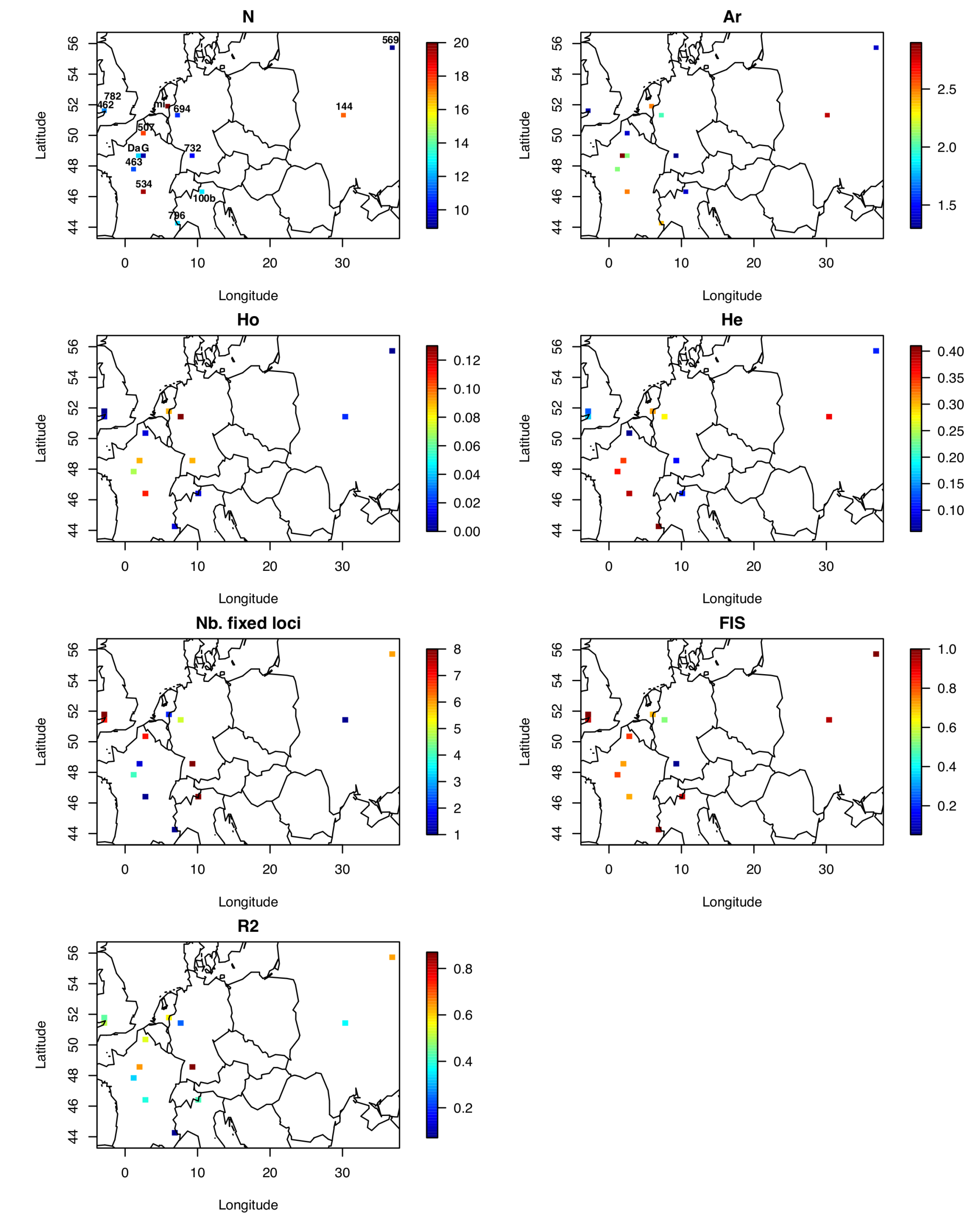

Supplement: Figure S11 — Mapped values overall loci for several descriptive statistics in Microbotryum lychnidis-dioicae (MvSl) population where the sample size was at least of 10 teliospores. The statistics shown include the sample size per locations (N), the mean allelic richness (Ar) per population, the mean observed and expected heterozygosity (Ho and He), the average number of fixed loci per populations (Nb. fixed loci), the overall loci inbreeding coefficient (FIS) per population, and the linkage disequilibrium across loci estimated using the correlation coefficient (r2) averaged across loci. We used the function quilt.plot in the R “Fields” package to plot the values on a map according to a grid system of 50×33 pixels. Locations that fall into the same grid box will have their values averaged. (0.75 MB TIF) [file ppat.1001229.s011.tif]

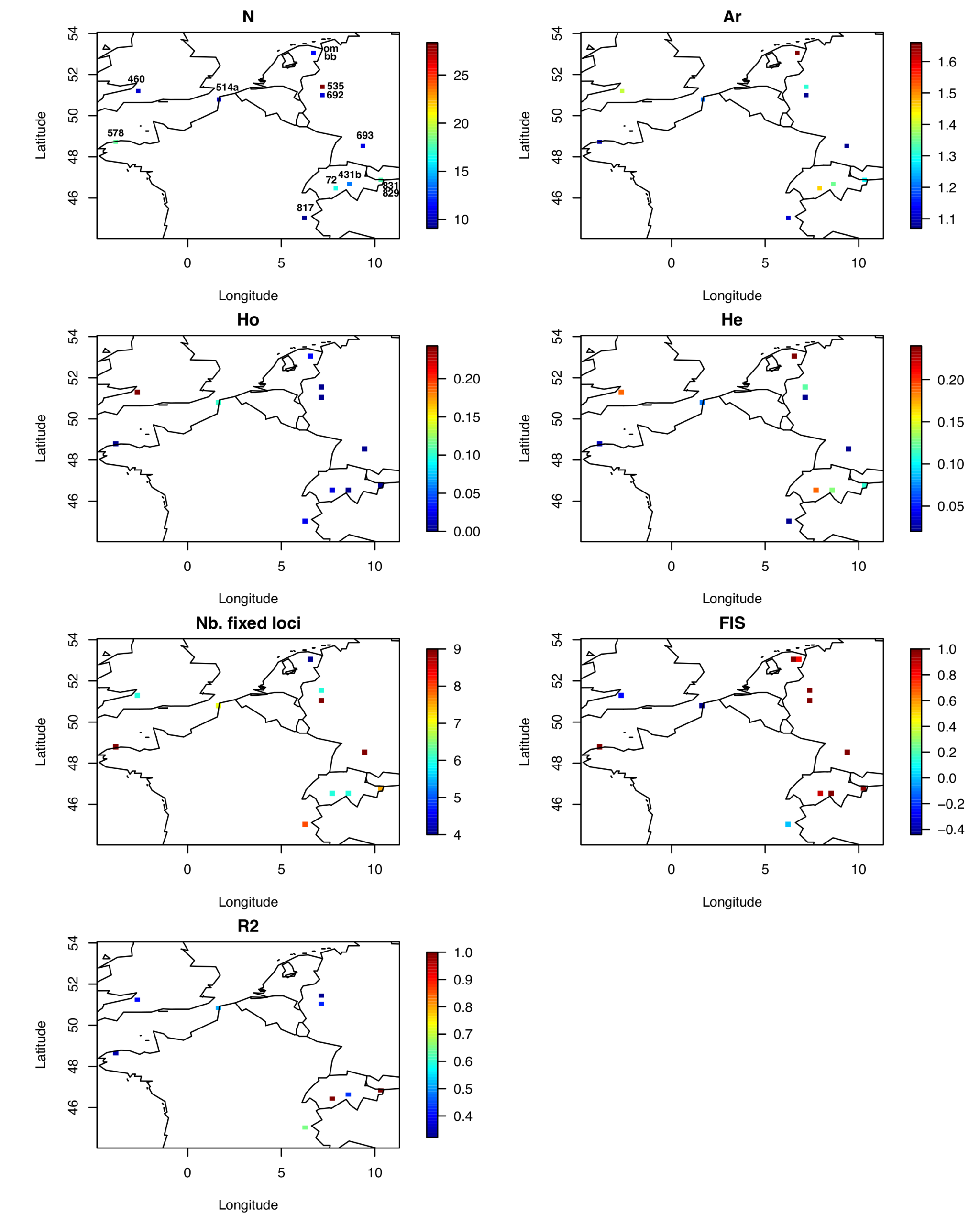

Supplement: Figure S12 — Same as Figure S11, but for Microbotryum silenes-dioicae (MvSd). The locus SL19 was excluded in the calculation of Ho, He, FIS, to avoid bias related to its almost complete fixation in a heterozygous state (see the text). (0.58 MB TIF) [file ppat.1001229.s012.tif]
