# Supplementary material for: Glacial Refugia in Pathogens: European Genetic Structure of Anther Smut Pathogens on Silene latifolia and Silene dioica
Source: PLoS Pathog. 2010 Dec 16;6(12):e1001229. doi: 10.1371/journal.ppat.1001229 (PMC3002987; doi:10.1371/journal.ppat.1001229)
Supplement: Text S1 — Description of genetic polymorphism in Microbotryum lychnidis-dioicae (MvSl) and M. silenes-dioicae (MvSd). (0.07 MB PDF) [file ppat.1001229.s013.pdf]

Text S1. Description of the genetic polymorphism in *Microbotryum lychnidis-dioicae* (MvSl) and *M. silenes-dioicae* (MvSd).

Overall polymorphism. Microbotryum diploid teliospores were collected on *S. latifolia* and on *S. doica* across Europe (Figure S1) and were genotyped using 11 microsatellite markers. Hybrids and cross-species disease transmission between MvSl and MvSd identified in a previous study [52] were removed from the dataset. We analyzed here 701 MvSl individuals from 187 localities and 342 MvSd individuals from 68 localities. The descriptive statistics of polymorphism and deviation from Hardy-Weinberg Equilibrium (HWE) are shown in Table 1. The number of alleles per locus was higher in MvSl than in MvSd (on average $Ar = 11.4 \pm 4.1$ *vs.* $Ar = 6.4 \pm 3.2$, Wilcoxon Signed Rank (WSR) test for paired samples $P = 0.003$). Both species displayed much lower levels of heterozygosity than expected under HWE. MvSl displayed only 3% of heterozygous genotypes while 73% were expected under HWE. Accordingly, F_{IS} over all loci was very high: 0.81 (95% Confidence Intervals (CI): [0.66-0.91]). MvSd was also highly homozygous at all loci but 3 (SL8, SVG5 and SL19). SL8 and SVG5 did not depart significantly from HWE, whereas SL19 showed strong excess in heterozygosity compared to HWE ($P < 0.001$), with only 8% of homozygous genotypes (Table 1). This locus strongly impacted the overall F_{IS} value (0.27, CI:[-0.33-0.84]) *vs.* 0.72, CI:[0.47-0.87] when SL19 was left out of the analysis).

Local polymorphism. In order to further characterise the impact of the mating system on diversity and structure, we focused on the pattern of genetic polymorphism within sampled localities that contained at least 10 successfully genotyped specimens (i.e., 15 and 13 localities for MvSl and MvSd, respectively, Figure S1). The figures S11 and S12 show the mapped values of descriptive statistics of the genetic polymorphism by locality in the two species.

The 15 localities with at least 10 samples of MvSl had a mean sample size of 13.1 ± 3.9 genotyped teliospore samples. The genetic diversity was low, as shown by the mean allelic richness of 1.8 ± 0.5 [min: 1.0 – max: 3.9] alleles per locus and population (standardized for a minimum sample size of 6 once accounting for locus specific missing data), the mean gene diversity per population of 0.24 ± 0.13 [min: 0.0 – max: 0.39], and an average of 4 ± 3 loci fixed in each population, with at least one locus fixed in each population and up to 8 loci in three populations. The allele frequencies displayed significant linkage disequilibrium in some populations with a mean r^2 of 0.45 ± 0.22 [min: 0.07 – max: 0.87] per population. F_{IS} values were high, with a mean of 0.79 ± 0.25 per population, and 9 out of 15

populations displayed values above 0.80, except one with F_{IS} = 0.05. This indicates that in most populations observed heterozygosity (H_o) was much lower than expected under HWE (overall loci H_o=0.05 compared to H_e =0.24, with 6 loci having less than 1% of heterozygous genotypes over all populations). This heterozygote deficit was highly significant (P < 0.001). Interestingly, while almost all F_{IS} values at each locus varied across populations between -0.25 and 1, the SL19 locus displayed the highest range of variation, between -1 and 1, indicating complete fixation in either a heterozygous or homozygous state. Among-population differences in allelic frequencies, estimated using F_{ST} value over all loci, were also very high with F_{ST} = 0.65 CI:[0.60-0.70] and highly significant for all pairs of populations ($p < 10^{-4}$, Table S1).

Similarly, the 13 localities with at least 10 samples of MvSd displayed a very low level of genetic diversity (Figure S12). The mean allelic richness (based on a minimum sample size of 5) was 1.3 ± 0.2 [min: 1.0 – max: 5.1] alleles per locus and per population, the gene diversity was 0.14 ± 0.09 [min: 0.00 – max: 0.31] per population, and an average of 7 ± 2 fixed loci was found per population, with at least 3 and up to 10 fixed loci in some populations. The level of heterozygosity was very low compared with HWE (over all loci excluding SL19, H_o/H_e= 0.03/0.60), except for the locus SL19, which showed strong excess in heterozygosity (H_o/H_e=0.85/0.65). Accordingly, the average F_{IS} value per population varied between 0.04 ± 0.59 [-0.69 – 0.98] or 0.66 ± 0.56 [-0.44 – 1.00], depending on whether SL19 was considered or not. The allelic frequencies displayed significant linkage disequilibrium in some populations with r^2 = 0.56 ± 0.27 [0.32 – 1.00], p < 0.0001) per population. Differences in allelic frequencies among these populations were very high, as shown by the overall F_{ST} value of 0.78 CI:[0.68 – 0.86] or 0.83 CI:[0.77-0.88] over all loci, depending on whether SL19 was included or not) (P < 0.0001 for all pairwise comparisons but one, Table S2).
