# Supplementary material for: Glacial Refugia in Pathogens: European Genetic Structure of Anther Smut Pathogens on Silene latifolia and Silene dioica
Source: PLoS Pathog. 2010 Dec 16;6(12):e1001229. doi: 10.1371/journal.ppat.1001229 (PMC3002987; doi:10.1371/journal.ppat.1001229)
Supplement: Table S1 — Level of pairwise differentiation, estimated as FST value, among samples of M. lychnidis-dioicae where n was higher than 10 strains. All pairwise values were significantly different at p<0.0001. (0.05 MB PDF) [file ppat.1001229.s014.pdf]

Table S1

	694	732	463	507	534	Da	G	796	850	100b	mi	569	462	782	144
**694**															
**732**	**0.77**														
**463**	0.57	**0.73**													
**507**	**0.79**	**0.91**	0.64												
**534**	0.53	0.67	*0.13*	0.52											
**Da**	0.52	0.68	*0.22*	0.56	*0.07*										
**G**	0.64	**0.78**	*0.23*	**0.72**	*0.20*	*0.18*									
**796**	0.58	**0.71**	0.51	**0.74**	0.50	0.49	0.56								
**850**	**0.74**	**0.87**	**0.71**	**0.90**	0.70	0.68	**0.77**	0.67							
**100b**	**0.78**	**0.88**	**0.75**	**0.92**	0.69	0.67	**0.77**	**0.70**	**0.82**						
**mi**	0.43	**0.74**	0.49	**0.72**	0.45	0.44	0.56	0.53	**0.72**	**0.73**					
**569**	**0.73**	**0.89**	0.67	**0.89**	0.66	0.66	**0.75**	0.65	**0.83**	**0.90**	0.68				
**462**	0.67	**0.84**	0.45	**0.75**	*0.26*	*0.24*	0.53	0.61	**0.83**	**0.83**	0.52	**0.83**			
**782**	**0.74**	**0.89**	0.60	**0.85**	0.54	0.54	0.67	0.64	**0.87**	**0.89**	0.68	**0.90**	**0.72**		
**144**	0.56	**0.74**	0.51	**0.72**	0.52	0.53	0.56	0.54	0.69	**0.72**	0.52	*0.31*	0.65	**0.71**	
