# Supplementary material for: Glacial Refugia in Pathogens: European Genetic Structure of Anther Smut Pathogens on Silene latifolia and Silene dioica
Source: PLoS Pathog. 2010 Dec 16;6(12):e1001229. doi: 10.1371/journal.ppat.1001229 (PMC3002987; doi:10.1371/journal.ppat.1001229)
Supplement: Table S2 — Same as Table S1 but for Microbotryum silenes-dioicae (MvSd) populations. All pairwise values but one (*) were significantly different at p<0.0001. (0.05 MB PDF) [file ppat.1001229.s015.pdf]

Table S2

	578	817	692	693	om	bb	829	831	535	514a	72	431b	460
**578**													
**817**	**0.84**												
**692**	**0.90**	**0.92**											
**693**	**0.90**	**0.92**	**0.50**										
**om**	**0.83**	**0.80**	**0.71**	**0.74**									
**bb**	**0.79**	**0.76**	**0.64**	**0.69**	-0.01*								
**829**	**0.96**	**0.97**	**0.96**	**0.96**	**0.88**	**0.85**							
**831**	**0.83**	**0.78**	**0.76**	**0.76**	**0.71**	**0.67**	**0.80**						
**535**	**0.78**	**0.76**	**0.84**	**0.84**	**0.74**	**0.70**	**0.90**	**0.79**					
**514a**	**0.83**	**0.83**	**0.88**	**0.88**	**0.74**	**0.68**	**0.95**	**0.78**	**0.72**				
**72**	**0.73**	**0.71**	**0.81**	**0.81**	**0.68**	**0.62**	**0.90**	**0.77**	**0.66**	*0.31*			
**431b**	**0.83**	**0.82**	**0.83**	**0.83**	**0.70**	**0.64**	**0.92**	**0.77**	**0.70**	**0.47**	**0.41**		
**460**	**0.77**	**0.72**	**0.72**	**0.72**	**0.68**	**0.62**	**0.90**	**0.73**	**0.65**	**0.59**	**0.50**	**0.51**	
